# Supplementary material for: COVID-19 Infection during Pregnancy: Disruptions in Lipid Metabolism and Implications for Newborn Health
Source: Int J Mol Sci. 2023 Sep 7;24(18):13787. doi: 10.3390/ijms241813787 (PMC10531385; doi:10.3390/ijms241813787)
Supplement: Supplementary file 1 [file ijms-24-13787-s001.zip › Supplementary S2.pdf]

Table S1. The number of samples of three biological fluids (maternal blood plasma, cord blood plasma and amniotic fluid) taken from 234 patients at the stage of delivery, whose lipid was determined by HPLC-MS/MS.

	Control (n=59)	Ia group (n=41)	Ib group (n=67)	Ic group (n=67)
mother plasma	51	27	51	50
Cord plasma	47	28	50	49
amniotic fluid	50	27	52	50

Table S2. Maternal plasma, cord blood, and amniotic fluid lipids, with statistically significant changes in history of Covid 19 infection, compared with controls.

Lipid	Control	Infection	P
Maternal plasma, 1st trimester, positive ion mode			
LPC 20:4	8.6e5(6.3e5;1.2e6)	1.1e6(8.6e5;1.3e6)	0.03
OxLPC 18:3(OOO)	2.1e5(1.6e5;2.6e5)	2.6e5(2.2e5;3.1e5)	0.03
OxTG 16:0_18:1_16:1(CHO)	2.2e5(1.5e5;2.8e5)	1.5e5(1.3e5;2.3e5)	0.03
OxTG 18:0_18:1_16:1(COOH)	1.9e5(1.7e5;2.1e5)	2.1e5(1.9e5;2.5e5)	0.03
LPC O-16:0	1.7e5(1.4e5;2.2e5)	2.1e5(1.7e5;2.6e5)	0.03
PC O-20:1/18:1	2.5e6(1.7e6;3.8e6)	1.6e6(1.0e6;2.9e6)	0.04
Maternal blood plasma, 1st trimester, negative ion mode			
LPC 16:0	2.9e6(2.4e6;4.1e6)	3.9e6(2.9e6;4.4e6)	0.02
LPC 18:2	4.6e5(3.5e5;5.9e5)	5.3e5(4.7e5;6.3e5)	0.04
LPC 20:4	1.1e5(7.7e4;1.7e5)	1.6e5(1.1e5;1.9e5)	0.03
PI 16:0_16:0	6.4e4(4.1e4;9.4e4)	4.1e4(3.3e4;5.1e4)	0.009
PI 16:0_18:1	2.8e5(2.1e5;4.1e5)	2.1e5(1.6e5;2.7e5)	0.009
PI 18:0_18:2	9.2e5(7.7e5;1.1e6)	7.4e5(6.1e5;9.2e5)	0.03
PI 18:0_20:3	2.7e5(2.2e5;3.4e5)	2.0e5(1.7e5;2.6e5)	0.004
PI 18:0_20:4	1.5e6(1.2e6;1.8e6)	1.2e6(1.0e6;1.5e6)	0.04
PMeOH 18:0_22:4	5.7e4(4.5e4;6.4e4)	4.9e4(3.5e4;5.5e4)	0.01
SM d20:0/16:0	2.1e5(1.1e5;3.1e5)	1.0e5(3.9e4;2.7e5)	0.03
Maternal blood plasma, 2nd trimester, positive ion mode			
PE 18:0_20:1	5.4e5(3.4e5;8.6e5)	8.1e5(5.1e5;9.9e5)	0.04
SM d18:1/18:1	2.4e6(2.1e6;3.1e6)	2.3e6(2.0e6;2.6e6)	0.049
SM d18:1/18:3	5.8e6(5.2e6;6.3e6)	5.5e6(4.8e6;5.9e6)	0.03
SM d18:1/24:0	7.8e6(4.6e6;9.1e6)	9.6e6(5.8e6;1.3e7)	0.02
TG 14:1_18:2_18:3	2.6e5(1.9e5;3.4e5)	3.2e5(2.3e5;4.1e5)	0.03
TG 16:0_16:1_20:1	2.2e5(1.5e5;4.5e5)	3.43e5(2.03e5;7e5)	0.03
TG 16:1_18:1_18:4	1.1e5(8.3e4;1.4e5)	1.2e5(8.9e4;1.6e5)	0.04
TG 16:1_18:2_18:3	1.7e6(1.3e6;2.1e6)	1.9e6(1.6e6;2.6e6)	0.02
TG 18:0_18:3_18:4	1.6e5(1.1e5;1.9e5)	1.8e5(1.3e5;2.25e5)	0.04
Maternal blood plasma, 2nd trimester, negative ion mode			
LPC 20:4	1.1e5(7.7e4;1.7e5)	1.6e5(9.5e4;2.0e5)	0.03

LPE 16:0	7.3e4(5.8e4;9.8e4)	1.0e5(6.3e4;1.3e5)	0.01
LPE 18:1	2.9e4(1.7e4;5.2e4)	4.6e4(3.1e4;6.6e4)	0.01
LPE 20:4	4.6e4(3.5e4;5.9e4)	5.4e4(4.3e4;8.2e4)	0.01
LPE 22:6	5.9e4(3.6e4;8.2e4)	7.8e4(4.0e4;1.1e5)	0.04
MGDG 18:2_22:6	3.0e6(2.8e6;3.4e6)	3.2e6(2.9e6;3.4e6)	0.04
MGDG 20:4_22:6	1.5e4(4.1e3;2.5e4)	2.2e4(1.0 e4;3.8e4)	0.04
PC 16:1_18:2	7.6e5(6.6e5;8.6e5)	8.4e5(7.0e5;1.0e6)	0.01
PC 18:0_20:3	8.2e4(7.1e4;9.6e4)	7.4e4(6.5e4;8.3e4)	0.009
PI 16:0_18:1	2.8e5(2.1e5;4.1e5)	2.4e5(1.9e5;3.2e5)	0.03
PI 18:0_18:2	9.2e5(7.7e5;1.1e6)	7.4e5(6.0e5;9.9e5)	0.02
PI 18:0_20:3	2.7e5(2.2e5;3.4e5)	2.4e5(1.9e5;2.9e5)	0.04
PI 18:0_20:4	1.5e6(1.2e6;1.8e6)	1.2e6(1.0e6;1.7e6)	0.04
PC O-18:0/18:2	2.1e4(1.5e4;3.0e4)	3.2e4(1.7e4;7.0e4)	0.01
PC O-20:0/20:4	3.9e4(2.8e4;6.3e4)	5.9e4(4.0e4;7.6e4)	0.01
Maternal plasma, 3rd trimester, positive ion mode			
LPC 16:0	1.5e7(9.9e6;2.4e7)	2.4e7(1.6e7;3.2e7)	0.005
LPC 18:0	5.8e6(4.1e6;8.2e6)	8.2e6(5.4e6;1.0e7)	0.003
LPC 18:1	2.8e6(2.1e6;3.9e6)	3.6e6(2.8e6;5.0e6)	0.006
LPC 18:2	3.3e6(2.6e6;4.8e6)	4.0e6(3.4e6;5.4e6)	0.03
LPC 20:3	3.3e5(2.6e5;4.2e5)	4.1e5(3.2e5;5.5e5)	0.002
LPC 20:4	8.6e5(6.3e5;1.2e6)	1.3e6(9.6e5;1.6e6)	<0.001
LPC 22:6	4.9e5(4.1e5;6.8e5)	6.3e5(5.3e5;8.2e5)	0.001
OxLPC 18:3(OOO)	2.1e5(1.6e5;2.8e5)	2.9e5(2.4e5;3.8e5)	<0.001
OxTG 16:0_18:1_16:1(CHO)	2.2e5(1.5e5;2.8e5)	1.5e5(1.2e5;2.4e5)	0.03
LPC O-16:0	1.7e5(1.4e5;2.2e5)	2.1e5(1.8e5;2.8e5)	0.001
TG 16:1_18:1_18:4	1.1e5(8.3e4;1.4e5)	1.2e5(9.9e4;1.5e5)	0.03
TG 18:0_18:3_18:4	1.6e5(1.1e5;1.9e5)	1.8e5(1.3e5;2.6e5)	0.02
Maternal blood plasma, 3rd trimester, negative ion mode			
LPC 16:0	2.9e6(2.4e6;4.1e6)	3.7e6(3.3e6;4.8e6)	0.001
LPC 18:0	7.2e5(5.1e5;1.0e6)	9.3e5(7.6e5;1.4e6)	0.003
LPC 18:1	4.3e5(3.0e5;5.8e5)	4.9e5(3.6e5;7.3e5)	0.02
LPC 20:4	1.1e5(7.7e4;1.7e5)	1.8e5(1.3e5;2.3e5)	<0.001
LPC 22:6	3.9e4(2.9e4;5.9e4)	5.4e4(3.7e4;8.1e4)	0.02
LPE 16:0	7.3e4(5.8e4;9.8e4)	1.0e5(7.9e4;1.3e5)	<0.001
LPE 20:4	4.6e4(3.5e4;5.9e4)	6.3e4(4.2e4;8.6e4)	0.007
LPE 22:6	5.9e4(3.6e4;8.2e4)	7.9e4(5.6e4;1.3e5)	0.004
OxCL 18:1(OH)_18:1 (OH)_22:6_22:6	1.2e5(9.9e4;1.4e5)	1.0e5(8.8e4;1.2e5)	0.03
OxPC 18:0_18:4(OO)	2.2e4(1.2e4;4.5e4)	3.0e4(1.5e4;9.9e4)	0.03
OxPC 18:2_14:1(COOH)	1.2e6(1.0e6;1.5e6)	1.0e6(8.2e5;1.3e6)	0.01
OxPC 20:4_16:1(COOH)	4.3e6(3.6e6;4.9e6)	4.9e6(4.3e6;5.9e6)	0.01

PC O-16:0/22:4	3.5e5(6.0e4;4.5e5)	1.2e5(6.9e4;3.5e5)	0.01
Cord blood plasma, 1st trimester, positive ion mode			
LPC 20:3	6.3e5(5.0e5;7.3e5)	7.1e5(5.7e5;7.9e5)	0.04
PC 16:0_22:6	3.8e7(3.2e7;4.3e7)	4.3e7(3.8e7;4.8e7)	0.03
PC 18:0_22:6	1.5e7(1.3e7;1.8e7)	2.0e7(1.5e7;2.0e7)	0.02
PE 18:0_20:4	1.1e6(8.2e5;1.4e6)	8.6e5(6.2e5;1.1e6)	0.03
TG 14:0_18:2_22:6	8.0e5(5.7e5;1.0e6)	1.0e6(7.3e5;1.2e6)	0.049
TG 16:0_18:1_20:5	5.1e5(4.0e5;5.6e5)	5.8e5(4.5e5;6.9e5)	0.04
TG 16:0_18:2_22:6	5.1e6(4.2e6;6.5e6)	7.0e6(5.4e6;8.1e6)	0.01
TG 16:0_20:4_22:6	1.6e6(1.3e6;2.2e6)	2.4e6(1.5e6;2.8e6)	0.04
TG 16:1_18:2_22:6	1.3e6(9.0e5;1.5e6)	1.6e6(1.4e6;2.0e6)	0.02
TG 16:1_18:3_22:6	1.9e5(1.3e5;2.5e5)	2.6e5(1.8e5;3.1e5)	0.02
TG 18:0_18:4_20:5	1.4e5(1.0e5;1.7e5)	1.8e5(1.2e5;2.0e5)	0.03
TG 18:1_18:2_20:5	4.0e5(3.4e5;5.0e5)	5.2e5(3.8e5;6.0e5)	0.03
Cord blood plasma, 1st trimester, negative ion mode			
PC 16:0_22:6	6.0e6(5.1e6;7.1e6)	7.1e6(6.1e6;7.8e6)	0.03
PC 18:0_22:6	2.4e6(2.0e6;3.0e6)	3.2e6(2.5e6;3.5e6)	0.008
LPE O-18:0	6.9e4(3.8e4;1.2e5)	4.7e4(2.3e4;6.5e4)	0.049
PE P-16:0/20:4	2.9e5(1.9e5;4.1e5)	2.0e5(1.3e5;2.5e5)	0.02
SM d20:0/18:0	2.9e4(1e3;2.95e5)	1.0e3(1.0e3;4.0e4)	0.046
SM d20:0/20:1	4.29e5(1e3;7.93e5)	6.3e5(2.7e5;1.5e6)	0.04
SM d22:0/22:5	1.2e6(1.1e6;1.6e6)	1.6e6(1.3e6;1.8e6)	0.02
Cord blood plasma, 2nd trimester, positive ion mode			
LPC 16:0	6.9e5(5.5e5;8.8e5)	8.5e5(7.1e5;9.6e5)	0.01
LPC 18:0	7.1e6(6.3e6;8.1e6)	7.8e6(7.1e6;8.8e6)	0.01
LPE 22:6	2.9e5(2.4e5;3.3e5)	3.3e5(2.5e5;3.9e5)	0.03
PC 16:0_22:6	3.8e7(3.2e7;4.3e7)	4.4e7(3.8e7;4.9e7)	0.002
PC 18:0_20:4	7.26e7(6.64e7;8e7)	8.0e7(6.9e7;9.0e7)	0.03
PC 18:0_22:6	1.5e7(1.3e7;1.8e7)	1.8e7(1.5e7;2.1e7)	<0.001
PC 20:1_20:5	6.1e6(5.0e6;7.1e6)	7.0e6(6.2e6;7.9e6)	<0.001
TG 16:0_18:1_20:5	5.1e5(4.0e5;5.6e5)	5.6e5(4.6e5;6.5e5)	0.01
TG 16:0_18:1_22:5	5.1e6(4.1e6;5.5e6)	5.5e6(4.8e6;6.4e6)	0.01
TG 16:0_18:1_22:6	6.9e6(6.1e6;8.3e6)	7.8e6(6.6e6;9.7e6)	0.03
TG 16:0_18:2_20:4	7.1e6(5.6e6;8.0e6)	8.2e6(6.6e6;9.4e6)	0.02
TG 16:0_18:2_22:6	5.1e6(4.2e6;6.5e6)	6.0e6(4.5e6;8.5e6)	0.03
TG 16:0_20:4_22:6	1.6e6(1.3e6;2.2e6)	2.2e6(1.4e6;2.8e6)	0.007
TG 16:1_18:2_22:6	1.3e6(9.0e5;1.5e6)	1.5e6(1.1e6;2.2e6)	0.02
TG 16:1_20:4_22:6	4.2e5(3.0e5;5.7e5)	5.7e5(3.3e5;8.4e5)	0.03
TG 18:0_18:4_20:5	1.4e5(1.0e5;1.7e5)	1.7e5(1.2e5;2.2e5)	0.01
TG 18:1_18:1_22:6	2.2e6(1.9e6;2.5e6)	2.4e6(2.0e6;2.9e6)	0.04
TG 18:1_18:2_20:3	2.9e5(2.5e5;3.4e5)	3.2e5(2.8e5;3.5e5)	0.02
TG 18:1_18:2_20:5	4.0e5(3.4e5;5.0e5)	4.8e5(3.6e5;6.0e5)	0.02
TG 18:1_18:2_22:6	2.3e6(1.9e6;2.8e6)	2.9e6(2.1e6;3.4e6)	0.01
TG 18:1_20:4_20:5	1.6e5(1.2e5;2.0e5)	2.0e5(1.4e5;2.6e5)	0.01
TG 18:1_20:4_22:6	1.1e6(8.1e5;1.3e6)	1.2e6(9.1e5;1.8e6)	0.01

TG 18:2_20:4_22:6	8.0e5(6.0e5;1.1e6)	1.1e6(6.2e5;1.7e6)	0.02
Cord blood plasma, 2nd trimester, negative ion mode			
CL 18:0_18:0_20:4_22:6	2.5e5(1.1e5;3.9e5)	1.2e5(8.7e4;2.3e5)	0.009
LPC 18:0	1.8e6(1.6e6;2.0e6)	2.0e6(1.7e6;2.2e6)	0.03
LPC 22:6	2.9e5(2.3e5;3.8e5)	3.6e5(2.8e5;4.3e5)	0.02
LPE 22:6	5.2e5(4.1e5;6.0e5)	6.1e5(4.5e5;7.2e5)	0.02
OxCL 18:1_18:3(OOH)2_18:3(OOH)2_20:3	3.8e4(2.6e4;5.2e4)	5.0e4(3.6e4;6.6e4)	0.03
OxLPE 16:1(CHO)	2.9e4(2.3e4;4.0e4)	2.1e4(1.3e4;4.5e4)	0.047
OxPC 16:0_20:3(1O)	3.6e5(2.6e5;4.8e5)	4.1e5(3.4e5;5.3e5)	0.048
OxPC 20:2_16:1(COOH)	5.2e5(3.7e5;6.4e5)	6.0e5(4.8e5;6.9e5)	0.04
OxPC 22:4_16:1(Ke,OH)	2.7e4(7.3e3;1.2e5)	4.2e4(2.5e4;2.6e5)	0.04
OxPC 22:6_16:1(COOH)	1.8e5(1.6e5;2.0e5)	2.0e5(1.7e5;2.1e5)	0.009
PC 16:0_22:6	6.0e6(5.1e6;7.1e6)	6.9e6(5.9e6;8.1e6)	0.004
PC 18:0_22:6	2.4e6(2.0e6;3.0e6)	3.1e6(2.5e6;3.4e6)	0.001
PI 16:0_18:2	5.9e4(3.2e4;7.6e4)	7.3e4(5.6e4;8.9e4)	0.01
PC O-16:1/20:4	5.3e5(2.3e5;6.5e5)	6.1e5(4.4e5;7.7e5)	0.04
PC O-22:1/22:6	1.7e6(1.4e6;2.1e6)	2.1e6(1.8e6;2.4e6)	0.001
PE P-18:1/22:6	6.8e4(5.9e4;8.4e4)	7.7e4(6.7e4;9.2e4)	0.03
SM d22:0/22:5	1.2e6(1.1e6;1.6e6)	1.6e6(1.4e6;1.8e6)	0.001
Cord blood plasma, 3rd trimester, positive ion mode			
LPE 22:6	2.9e5(2.4e5;3.3e5)	3.2e5(2.4e5;3.8e5)	0.03
PC 18:1_20:5	1.0e7(7.9e6;1.3e7)	1.2e7(9.4e6;1.4e7)	0.049
TG 16:0_18:1_22:6	6.9e6(6.1e6;8.3e6)	8.0e6(6.5e6;9.4e6)	0.04
TG 16:0_20:4_22:6	1.6e6(1.3e6;2.2e6)	1.9e6(1.4e6;2.6e6)	0.04
TG 16:1_20:4_22:6	4.2e5(3.0e5;5.7e5)	5.0e5(3.9e5;7.5e5)	0.04
TG 18:0_18:1_18:1	2.1e6(1.4e6;3.86)	1.4e6(1.1e6;2.5e6)	0.03
TG 18:0_18:4_20:5	1.4e5(1.0e5;1.7e5)	1.5e5(1.2e5;1.9e5)	0.03
TG 18:1_18:1_22:6	2.2e6(1.9e6;2.5e6)	2.4e6(2.1e6;2.8e6)	0.02
TG 18:1_18:2_22:6	2.3e6(1.9e6;2.8e6)	2.7e6(2.1e6;3.3e6)	0.02
TG 18:1_20:4_22:6	1.1e6(8.1e5;1.3e6)	1.2e6(8.9e5;1.7e6)	0.03
TG 18:2_20:4_22:6	8.0e5(6.0e5;1.1e6)	9.8e5(7.3e5;1.4e6)	0.03
Cord blood plasma, 3rd trimester, negative ion mode			
CL 18:0_18:0_20:4_22:6	2.5e5(1.1e5;3.9e5)	1.2e5(9.9e4;3.0e5)	0.047
LPC 18:2	1.7e6(1.2e6;2.4e6)	2.1e6(1.4e6;3.0e6)	0.02
LPC 20:3	5.1e5(3.6e5;6.1e5)	6.1e5(4.3e5;7.3e5)	0.04
LPC 20:5	2.6e4(2.0e4;3.8e4)	3.2e4(2.4e4;5.5e4)	0.048
LPC 22:6	2.9e5(2.3e5;3.8e5)	3.7e5(2.7e5;4.3e5)	0.01
LPE 18:2	1.9e5(1.5e5;2.4e5)	2.2e5(1.7e5;2.6e5)	0.04
LPE 20:4	5.0e5(4.2e5;6.0e5)	5.9e5(4.8e5;7.0e5)	0.008
LPE 22:6	5.2e5(4.1e5;6.0e5)	6.5e5(5.1e5;7.6e5)	0.001
OxPC 20:2_16:1(COOH)	5.2e5(3.7e5;6.4e5)	5.9e5(4.5e5;7.6e5)	0.02
OxPC 22:6_16:1(COOH)	1.8e5(1.6e5;2.0e5)	2.0e5(1.7e5;2.1e5)	0.04
OxPE 18:2_16:1(COOH)	3.2e5(2.0e5;4.3e5)	3.9e5(2.8e5;5.1e5)	0.045
PC 16:0_20:4	9.3e6(5.8e6;1.2e7)	1.0e7(8.5e6;1.2e7)	0.04
PEtOH 16:0_22:4	3.8e4(2.3e4;8.1e4)	2.3e4(9.9e3;5.6e4)	0.047
PC O-16:0/20:4	1.3e5(9.8e4;1.6e5)	1.6e5(1.3e5;2.0e5)	0.006

PC O-22:1/22:6	1.7e6(1.4e6;2.1e6)	1.9e6(1.6e6;2.3e6)	0.04
PC O-24:0/20:4	5.3e5(2.7e5;8.0e5)	7.6e5(5.5e5;9.9e5)	0.005
PE P-18:1/22:6	6.8e4(5.9e4;8.4e4)	7.6e4(6.6e4;9.7e4)	0.04
SM d20:1/16:1	2.9e4(1.0e3;5.0e4)	5.3e4(1.0e3;1.4e5)	0.02
SM d24:1/18:1	7.6e4(3.8e4;1.0e5)	8.6e4(6.8e4;1.2e5)	0.02
Amniotic Fluid 1st Trimester Positive Ion Mode			
PC O-16:0/20:4	8.1e5(5.3e5;1.2e6)	1.1e6(7.7e5;2.0e6)	0.045
Amniotic Fluid 1st Trimester Negative Ion Mode			
PI 18:1_20:4	1.5e5(9.0e4;3.1e5)	2.8e5(1.9e5;5.2e5)	0.04
PE P-20:0/18:2	3.0e4(2.0e4;7.1e4)	5.4e4(3.4e4;1.3e5)	0.01
Amniotic Fluid 2nd Trimester Positive Ion Mode			
PC 16:0_18:1	6.6e7(4.7e7;9.3e7)	5.0e7(2.2e7;7.9e7)	0.03
SM d22:5/22:1	5.8e5(3.6e5;1.1e6)	1.0e6(5.8e5;1.5e6)	0.049
TG 16:0_22:2_26:0	5.1e5(3.1e5;1.1e6)	4.2e5(2.8e5;5.8e5)	0.03
Amniotic Fluid 2nd Trimester Negative Ion Mode			
LPG 16:0	1.6e5(1.0e3;5.4e5)	2.9e4(1.0e3;1.6e5)	0.03
PC 18:1_18:1	1.1e6(5.5e5;2.9e6)	4.8e5(1.0e3;2.2e6)	0.04
PI 16:0_18:1	4.5e5(2.2e5;8.7e5)	2.1e5(5.3e4;7.4e5)	0.01
PI 18:1_22:5	2.0e5(1.1e5;3.2e5)	1.3e5(5.8e4;2.6e5)	0.03
Amniotic Fluid 3rd Trimester Positive Ion Mode			
PC 14:1_16:0	3.9e6(2.0e6;7.0e6)	3.1e6(1.4e6;4.5e6)	0.049
PG 18:0_18:1	5.6e5(3.8e5;8.1e5)	3.5e5(2.2e5;5.4e5)	0.002
PC O-18:0/16:1	6.1e5(3.1e5;9.2e5)	8.9e5(4.2e5;1.5e6)	0.04
SM d22:6/18:2	5.1e5(4.3e5;7.6e5)	4.5e5(3.3e5;6.6e5)	0.02
Amniotic Fluid 3rd Trimester Negative Ion Mode			
LPG 16:0	1.6e5(1.0e3;5.4e5)	6.7e3(1.0e3;1.6e5)	0.03
MGDG 16:0_18:0	1.7e4(5.5e3;3.0e4)	2.6e4(1.8e4;1.2e5)	0.006
PC 18:1_18:1	1.1e6(5.5e5;2.9e6)	9.1e5(1.0e3;1.8e6)	0.02

Table S3. OPLS-model quality characteristic.

Task	R^2X	R^2Y	Q^2Y
Health child, control group	0.35	0.29	-0.42
Health child, the 1-st trimester group	0.41	0.51	-0.32
Health child, the 2-nd trimester group	0.41	0.30	-0.45
Health child, the 3-rd trimester group	0.42	0.32	-0.54
CHD, the control group	0.35	0.35	-0.21
CHD, the 2-nd trimester group	0.41	0.26	-0.34

Table S4. Model variables for determining whether a child will be healthy in the control group according to the plasma metabolomic profile

Variable	VIP (OPLS)	β	CI β	Wald criterion	P
Free term	-	21.57	10.21 - 42.35	2.80	0.005
PC 16:0_16:0 *TG 14:0_16:1_18:2	1.13	$-5.43*10^{-12}$	$-1.09*10^{-11}$ - $-2.58*10^{-12}$	-2.75	0.006
OxLPC 18:4(OOO) * PC 16:0_18:2	1.03	$-5.91*10^{-13}$	$-1.20*10^{-12}$ - $-2.60*10^{-13}$	-2.63	0.009

PC P-16:0/18:2 * TG 16:0_16:0_18:1	1.45	$3.85*10^{-13}$	$1.61*10^{-13}$ - $8.01*10^{-13}$	2.52	0.01
PC 16:0_20:5 * OxPC 18:2_16:1(COOH)	1.51	$-1.88*10^{-14}$	$-3.89*10^{-14}$ - $-6.57*10^{-15}$	-2.42	0.02

Table S5. Model variables for determining whether a baby will be healthy in a cohort of patients infected with COVID-19 during the first trimester of pregnancy by plasma metabolomic profile.

Variable	VIP (OPLS)	β	CI β	Wald criterion	P
Free term	-	4.53	0.97 - 10.77	1.93	0.05
PC 16:1_18:0 * LPC 16:1	1.31	$-1.17*10^{-12}$	$-2.56*10^{-12}$ - $-3.88*10^{-13}$	-2.24	0.03

Table S6. Model variables for determining whether a baby will be healthy in a cohort of patients infected with COVID-19 during the second trimester of pregnancy by plasma metabolomic profile.

Variable	VIP (OPLS)	β	CI β	Wald criterion	P
Free term	-	15.22	6.97 - 28.60	2.80	0.005
PC 16:0_20:1 * TG 16:0_16:1_18:0	1.90	$-2.95*10^{-13}$	$-5.67*10^{-13}$ - $-1.27*10^{-13}$	-2.69	0.007
SM d18:0/22:0 * TG 18:1_22:5_8:0	1.56	$-2.09*10^{-13}$	$-3.82*10^{-13}$ - $-9.74*10^{-14}$	-2.93	0.003
MGDG 16:0_20:0 * SM d18:0/22:0	1.23	$4.48*10^{-13}$	$1.32*10^{-13}$ - $9.27*10^{-13}$	2.28	0.02
PC 18:1_18:3 * OxPC 18:2_16:1(COOH)	4.48	$-3.36*10^{-14}$	$-6.84*10^{-14}$ - $-9.99*10^{-15}$	-2.31	0.02

Table S7. Model variables for determining whether a baby will be healthy in a cohort of patients infected with COVID-19 during the third trimester of pregnancy by plasma metabolomic profile.

Variable	VIP (OPLS)	β	CI β	Wald criterion	P
Free term	-	6.22	1.88 - 12.82	2.33	0.02
SM d18:1/24:1 * PC 18:1_20:1	2.88	$-2.01*10^{-13}$	$-4.05*10^{-13}$ - $-3.00*10^{-14}$	-2.55	0.01
PC 16:0_22:6 * PC O-16:0/20:4	1.59	$-5.41*10^{-13}$	$-1.04*10^{-12}$ - - $2.39*10^{-13}$	-2.74	0.006
PC 16:1_22:6 * PC 16:0_22:6	1.32	2.36*10-^{13}	$9.42*10^{-14}$ -$4.67*10^{-13}$	2.59	0.01
LPC 16:0 * PC P-20:1/18:1	1.56	$-6.50*10^{-14}$	$-1.39*10^{-13}$ - $-2.00*10^{-14}$	-2.15	0.03

Table S8. Model variables for determining CHD in a child, in a control group of patients according to the plasma metabolomic profile.

Variable	VIP (OPLS)	β	CI β	Wald criterion	P
Free term	-	-20.04	-42.68 - -7.67	-2.29	0.02
PC 18:2_18:2 * OxCL 18:2_22:6_18:3_22:5(OOH)2	2.96	$3.33*10^{-13}$	$1.32*10^{-13}$ -- $6.98*10^{-13}$	2.36	0.02
PC 18:0_18:2 * PC O-18:0/18:1	1.10	-1.81*10-^{13}	$-3.90*10^{-13}$ - $-6.21*10^{-14}$	-2.31	0.02

Table S9. Model variables for the determination of CHD in a child, in a group of patients infected with COVID-19 during the second trimester of pregnancy according to the plasma metabolomic profile.

Variable	VIP (OPLS)	β	CI β	Wald criterion	P
Free term	-	4.26	0.14 - 9.98	1.77	0.08
CE 16:0 * TG 18:1_22:5_8:0	1.10	$3.42*10^{-13}$	$1.70*10^{-13}$ - $6.01*10^{-13}$	3.22	0.001
LPC 16:0 * PE 18:0_20:1	1.01	$-1.65*10^{-13}$	$-3.01*10^{-13}$ - $-6.79*10^{-14}$	-2.86	0.004
PC 16:0_20:3 * TG 18:1_18:1_20:1	1.77	$-8.28*10^{-14}$	$-1.77*10^{-13}$ - $-1.86*10^{-14}$	-2.06	0.04
PC 16:0_16:0 * SM d18:1/18:3	1.26	$-1.31*10^{-13}$	$-2.63*10^{-13}$ - $-3.11*10^{-14}$	-2.29	0.02

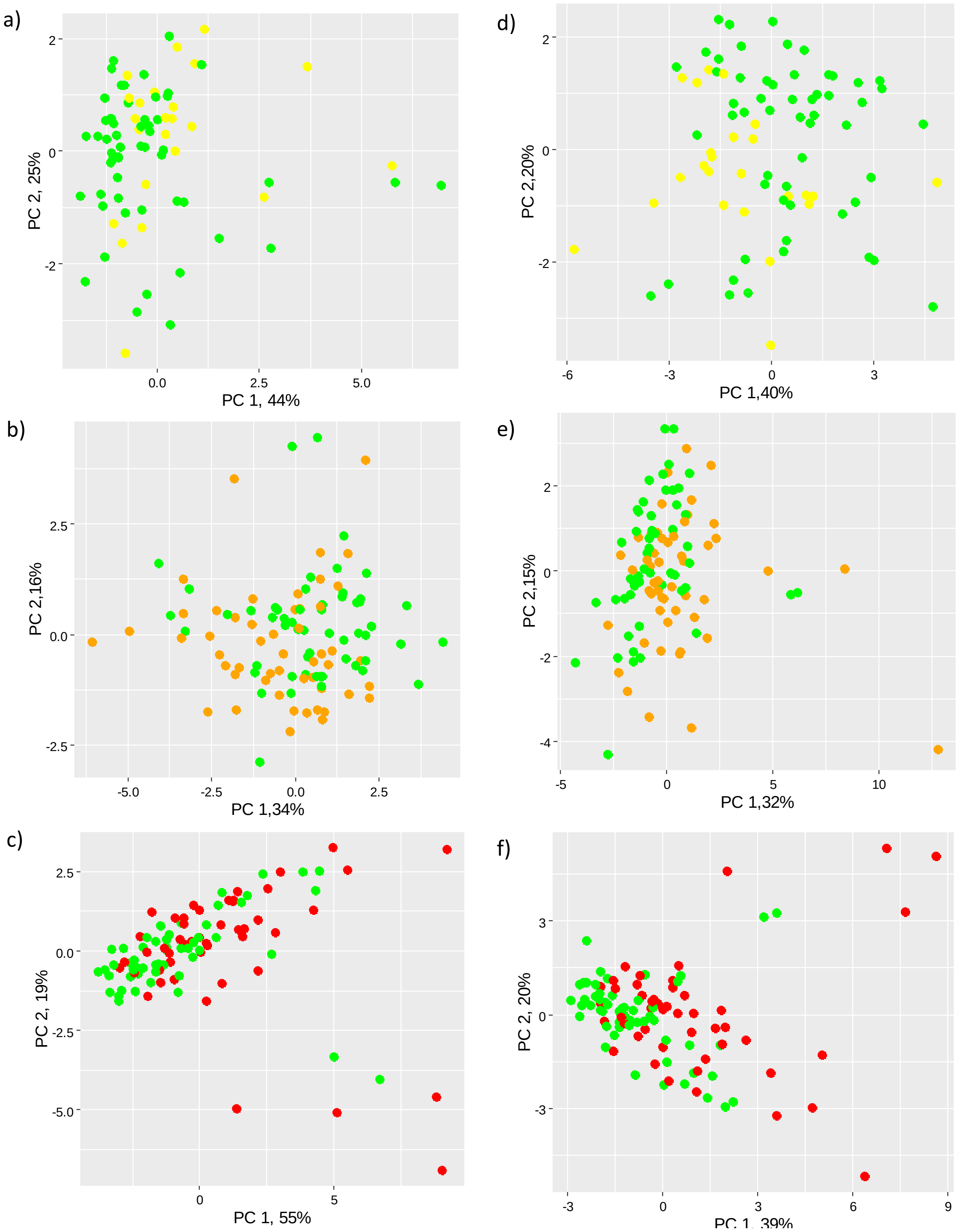

Figure S1. Maternal venous plasma samples in principal component spaces, which are based on statistical significantly differences lipid between two groups. a)-c) – positive ion mode, d)-f) – negative ion mode. Green – control group samples, yellow – covid during the 1st trimester group sample, orange - covid during the 2nd trimester group sample, red - covid during the 3rd trimester group sample.

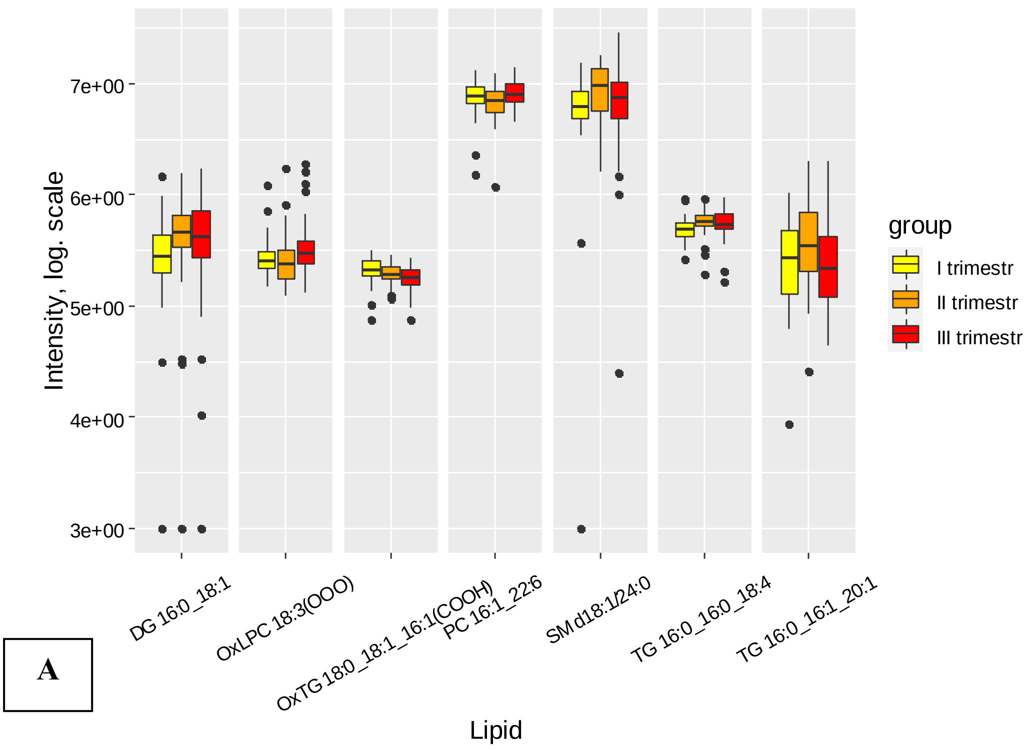

A

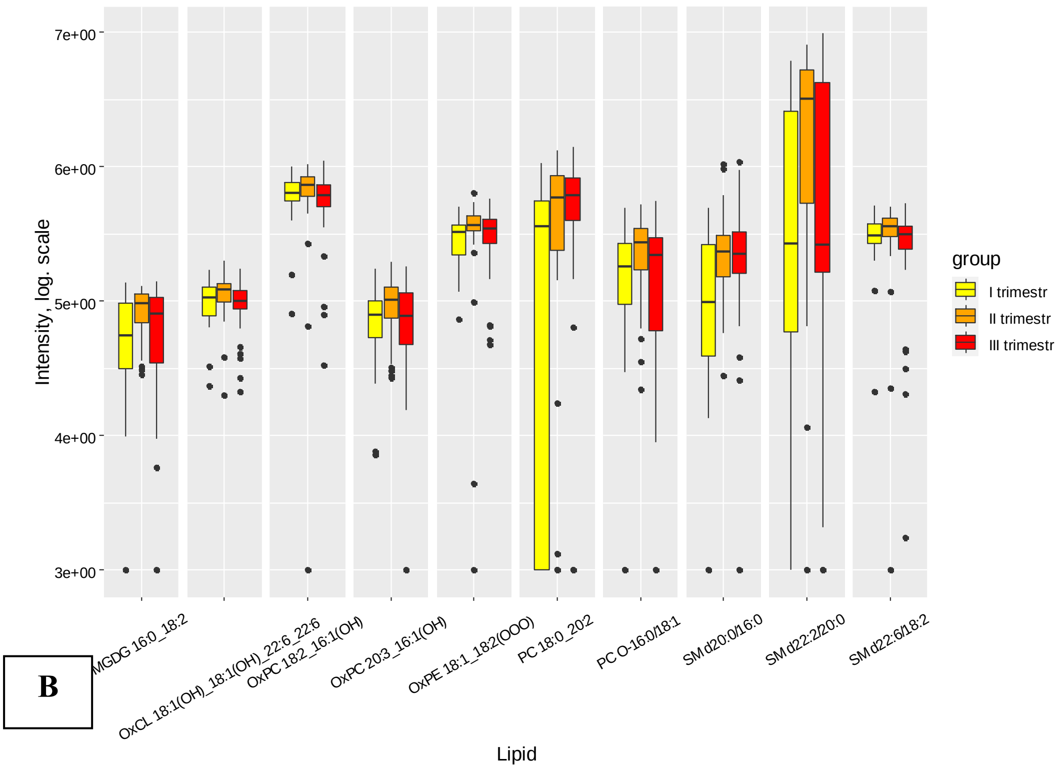

B

Figure S2. Lipids characterizing differences in the time of infection in maternal plasma (mode of positive (A) and negative (B) ions).

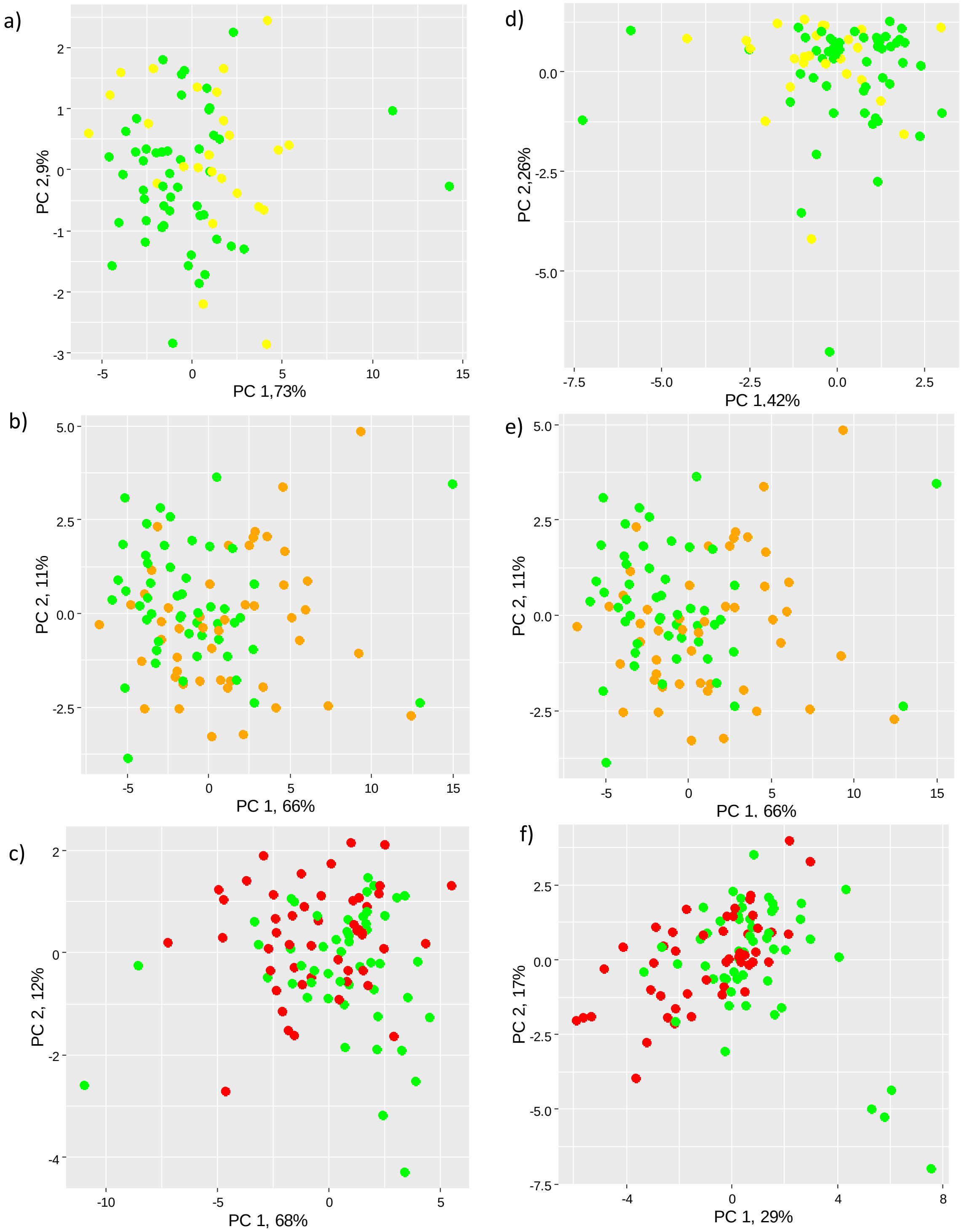

Figure S3. Cord plasma samples in principal component spaces, which are based on statistical significantly differences lipid between two groups. a)-c) – positive ion mode, d)-f) – negative ion mode. Green – control group samples, yellow – covid during the 1st trimester group sample, orange - covid during the 2nd trimester group sample, red - covid during the 3rd trimester group sample.

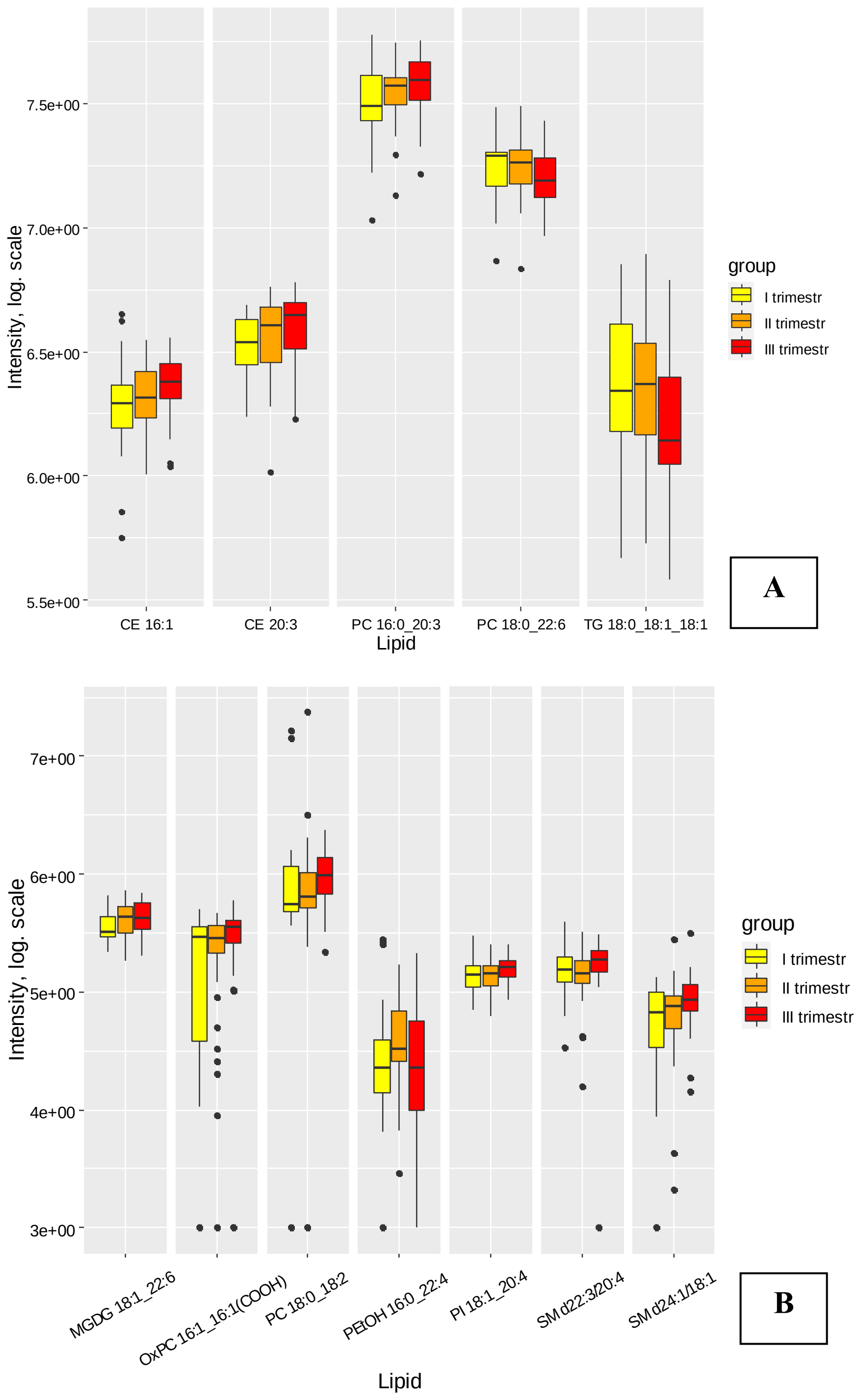

Figure S4. Lipids characterizing differences in the time of infection in umbilical cord plasma (mode of positive (A) and negative (B) ions).

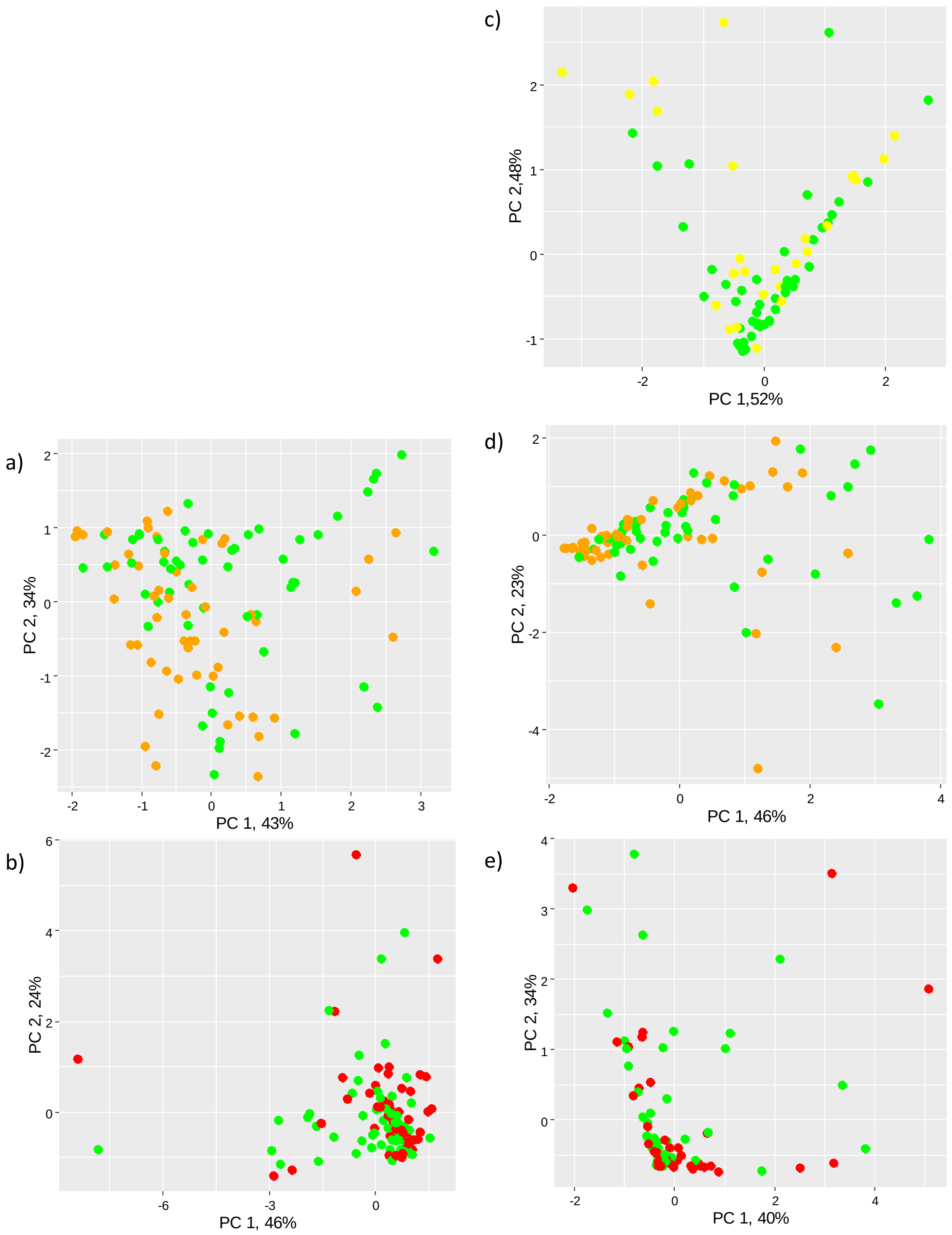

Figure S5. Amniotical fluid samples in principal component spaces, which are based on statistical significantly differences lipid between two groups. a)-c) – positive ion mode, d)-f) – negative ion mode. Green – control group samples, yellow – covid during the 1st trimester group sample, orange - covid during the 2nd trimester group sample, red - covid during the 3rd trimester group sample.
