# Supplementary material for: COVID-19 Infection during Pregnancy: Disruptions in Lipid Metabolism and Implications for Newborn Health
Source: Int J Mol Sci. 2023 Sep 7;24(18):13787. doi: 10.3390/ijms241813787 (PMC10531385; doi:10.3390/ijms241813787)

Maternal plasma, positive ion mode

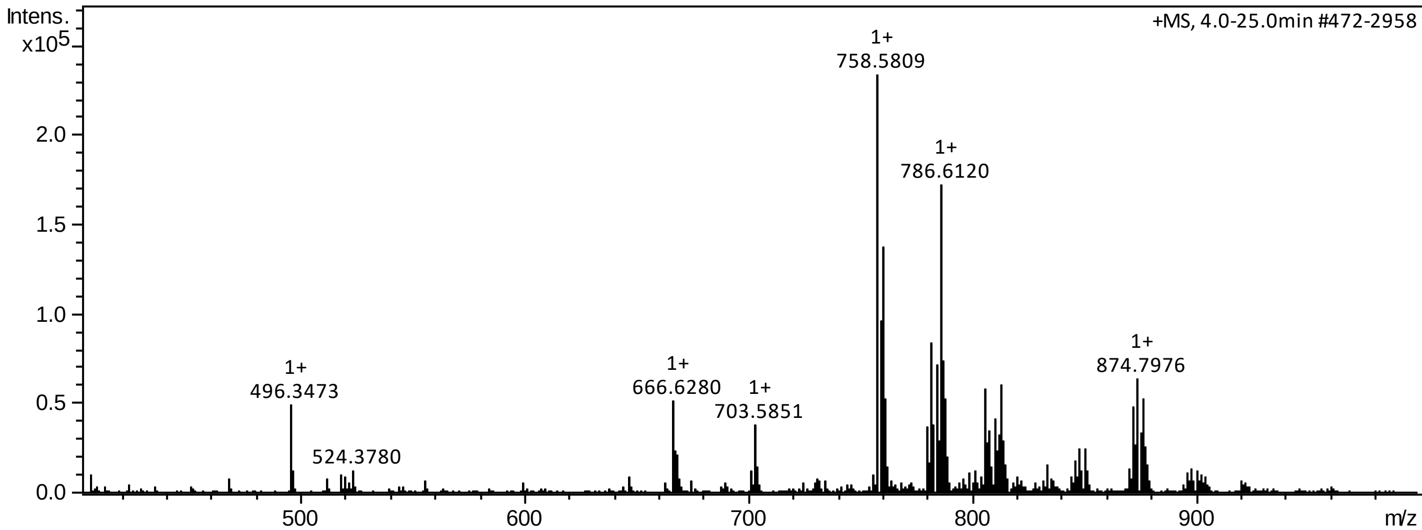

Maternal plasma, negative ion mode

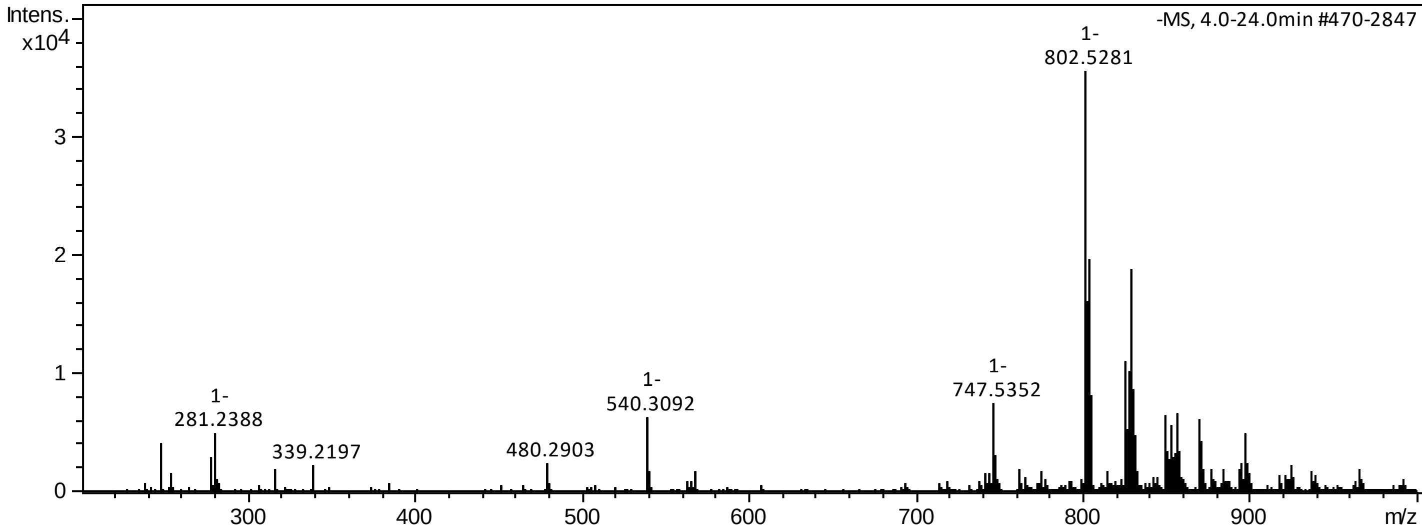

Cord plasma, positive ion mode

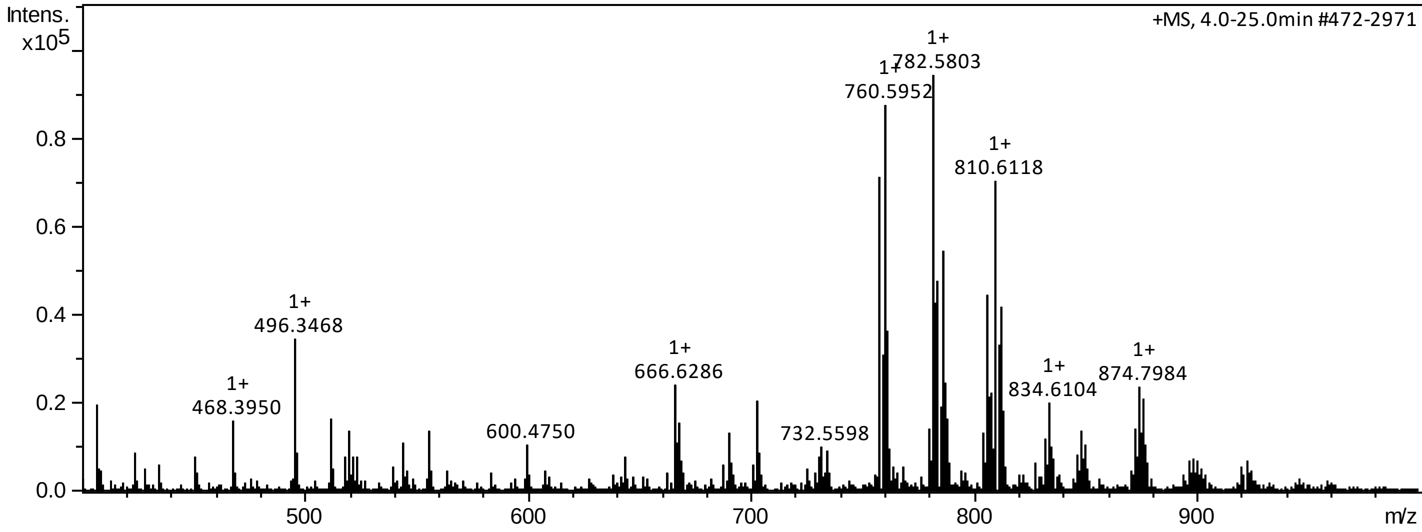

Cord plasma, negative ion mode

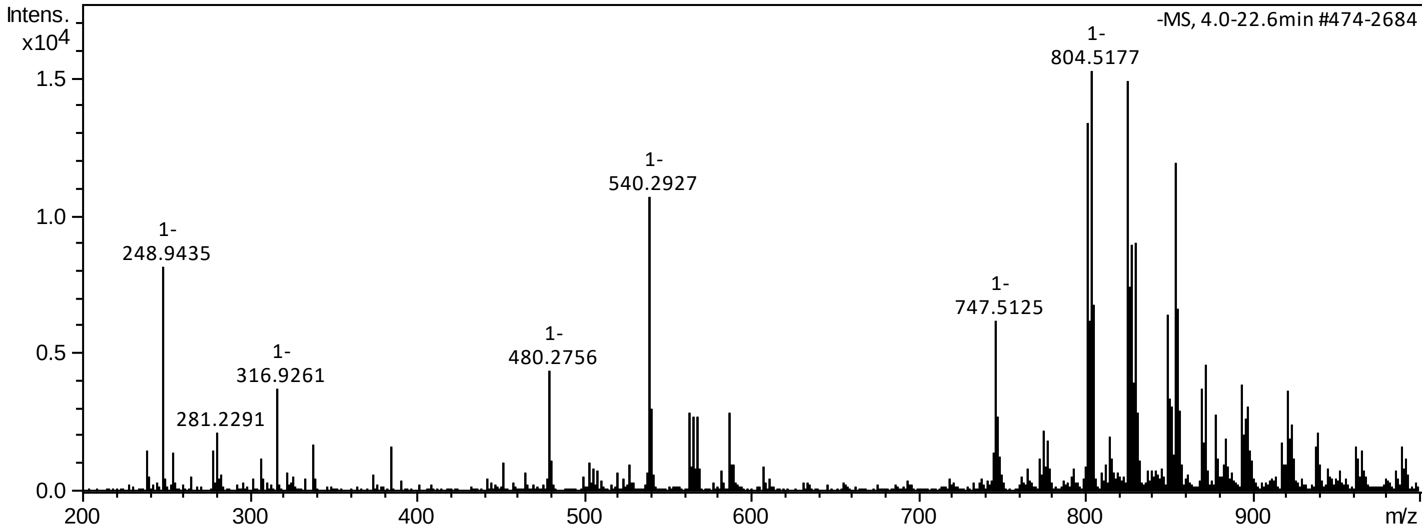

Amniotic fluid, positive ion mode

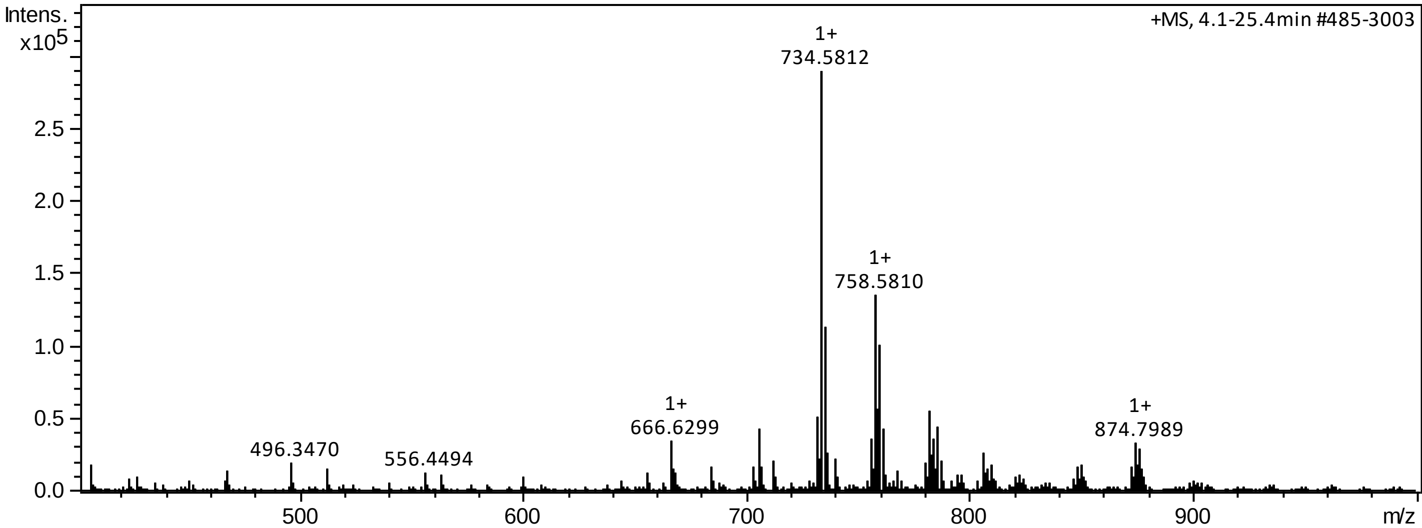

Amniotic fluid, negative ion mode

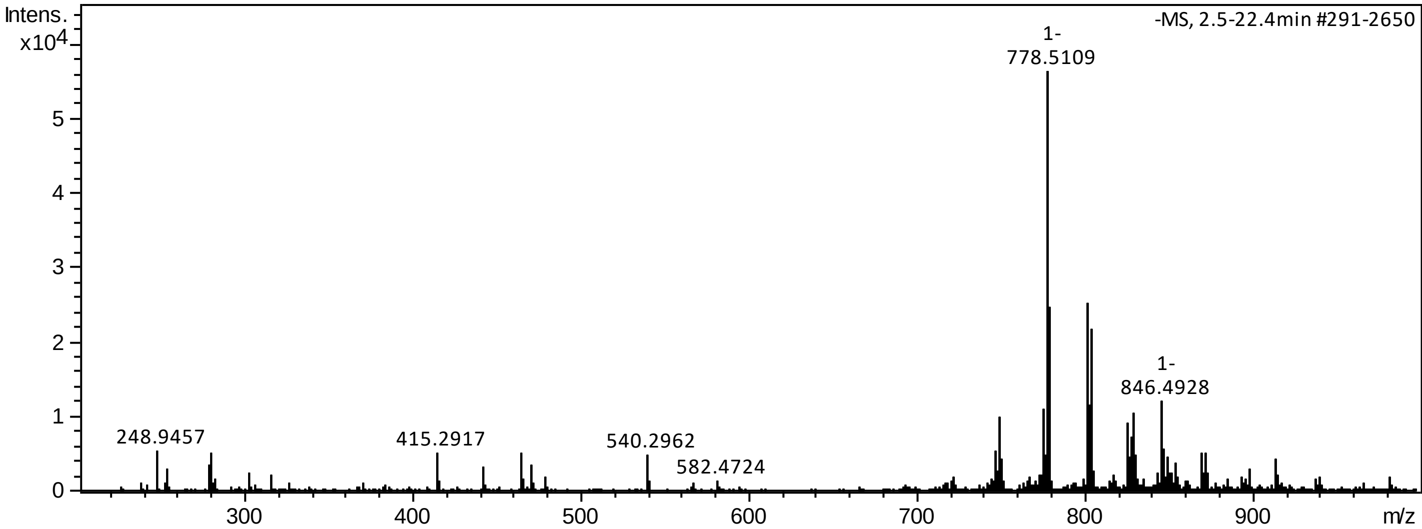

Supplement: Supplementary file 1 [file ijms-24-13787-s001.zip › Supplementary S4.pdf]
