# Supplementary material for: COVID-19 Infection during Pregnancy: Disruptions in Lipid Metabolism and Implications for Newborn Health
Source: Int J Mol Sci. 2023 Sep 7;24(18):13787. doi: 10.3390/ijms241813787 (PMC10531385; doi:10.3390/ijms241813787)

CE 16:0

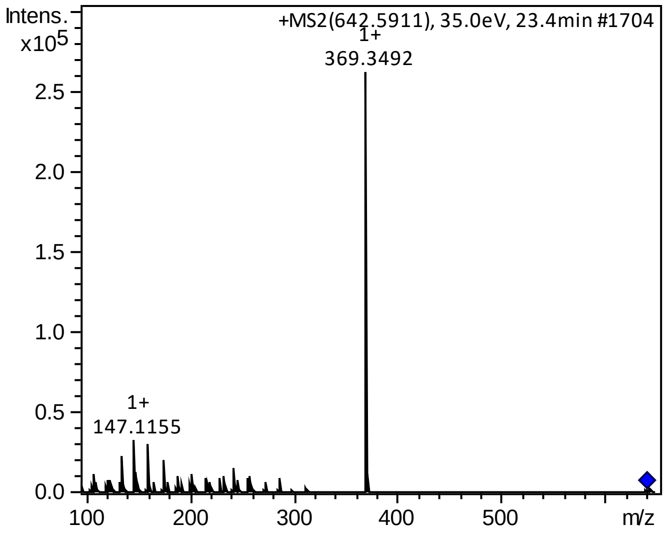

LPC 16:0

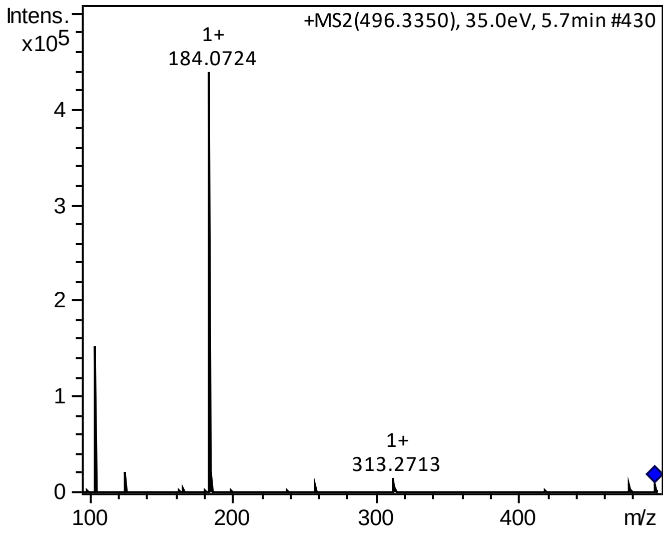

LPC 16:1

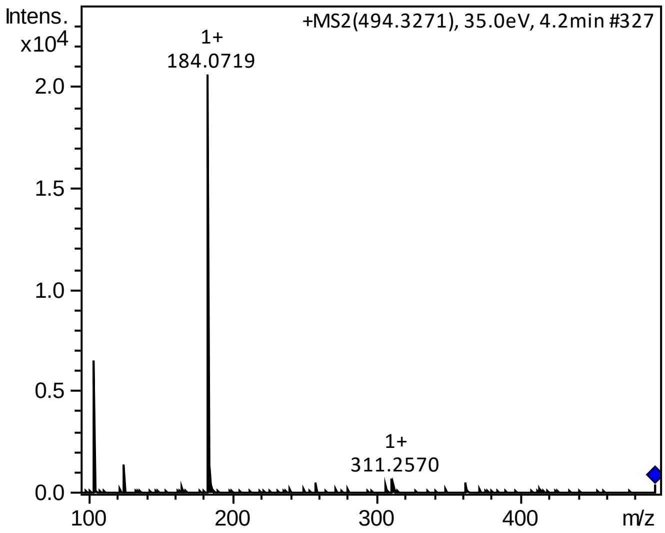

MGDG 16:0_20:0

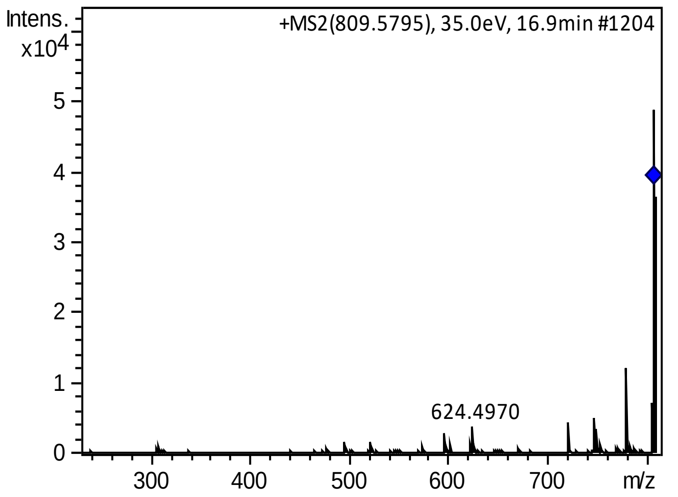

OxCL 18:2_22:6_18:3_22:5(OOH)2

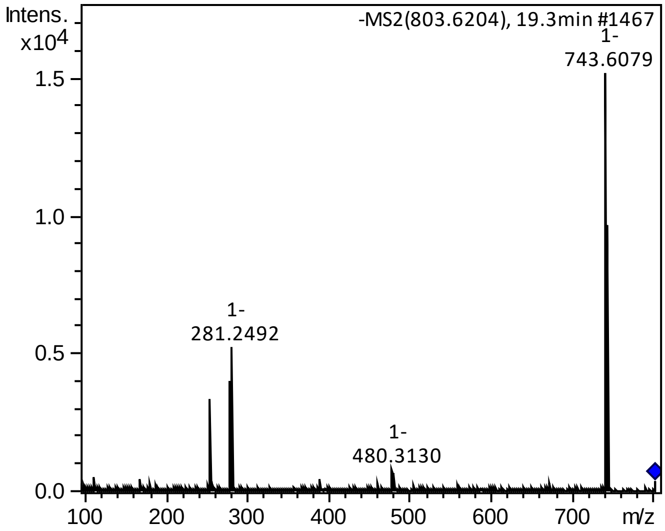

OxLPC 18:4(OOO)

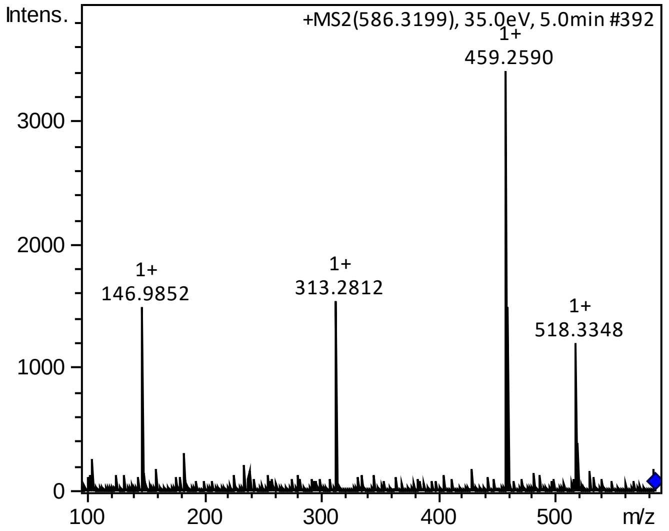

OxPC 18:2_16:1(COOH)

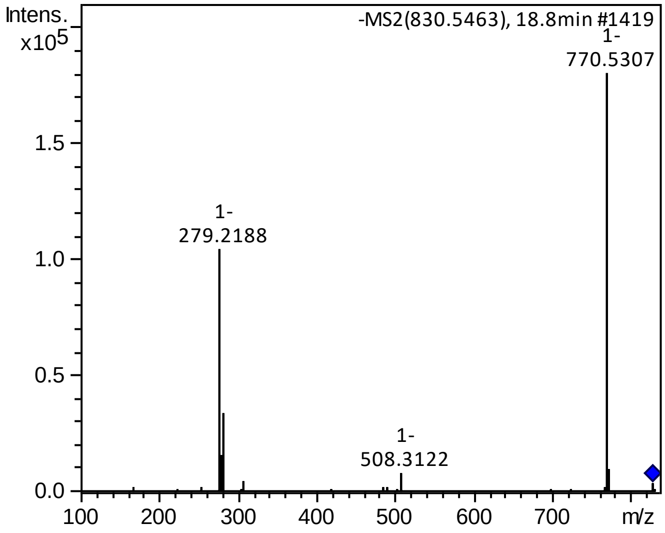

PC 16:0_16:0

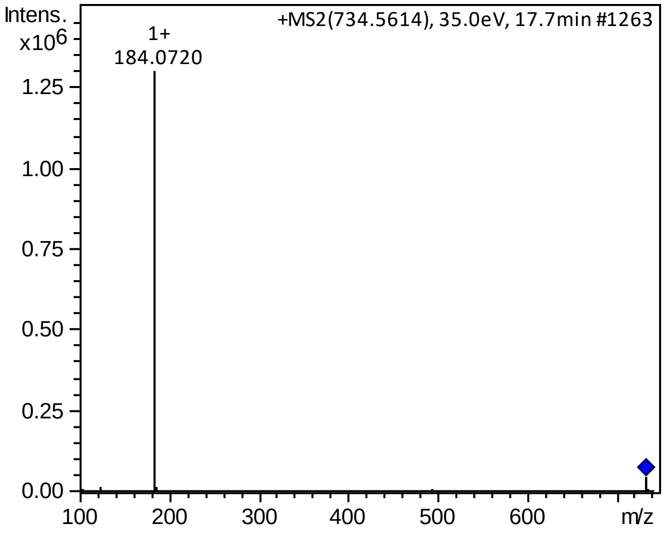

PC 16:0_18:2

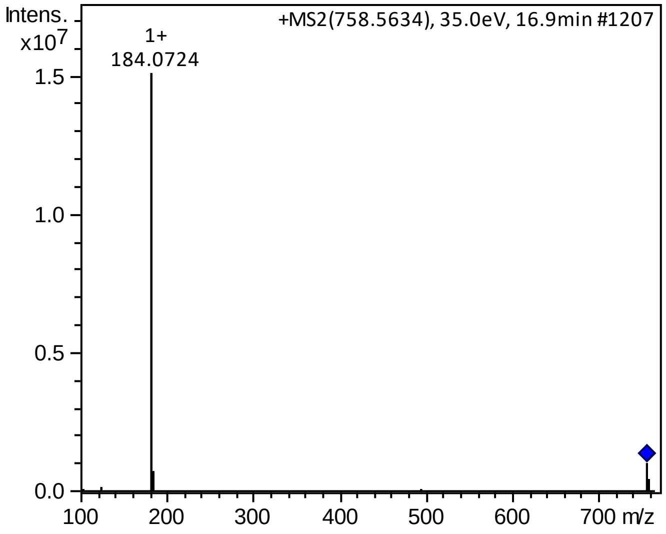

PC 16:0_20:1

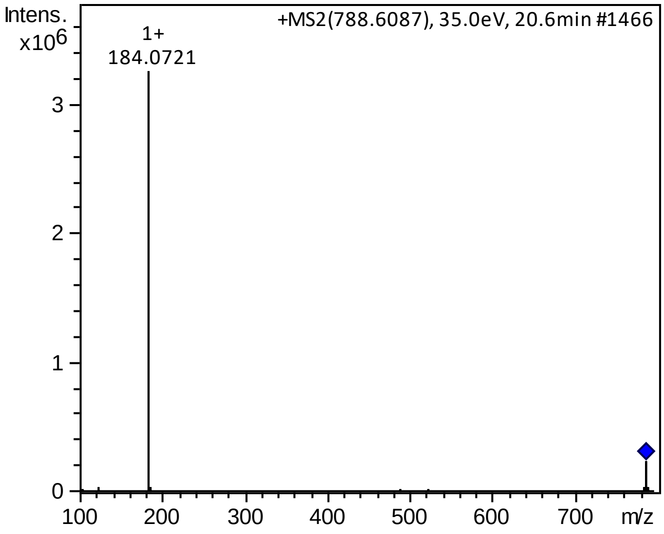

PC 16:0_20:3

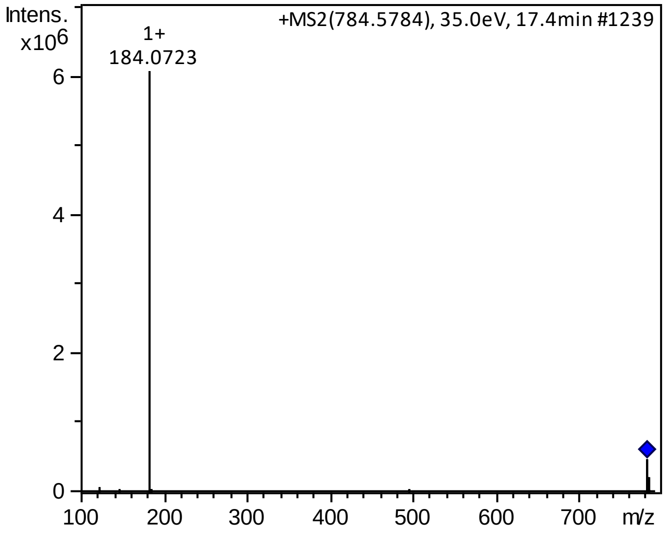

PC 16:0_20:5

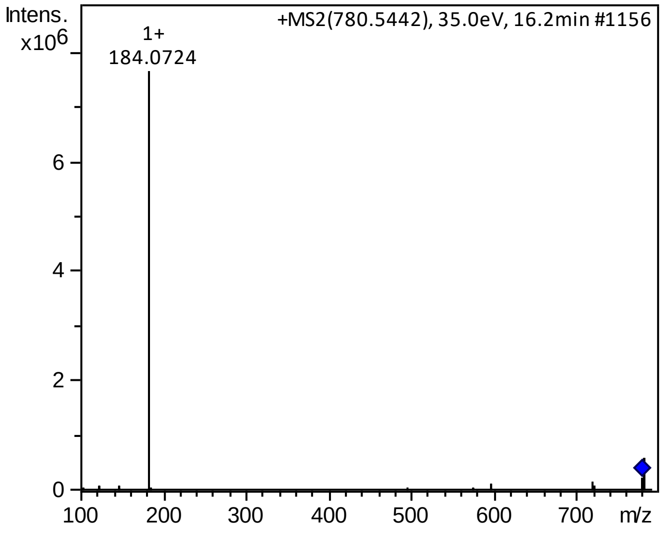

PC 16:0_22:6

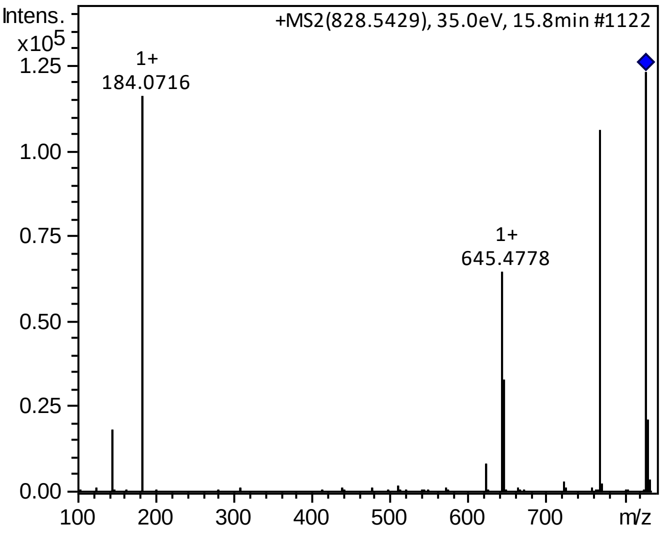

PC 16:1_18:0

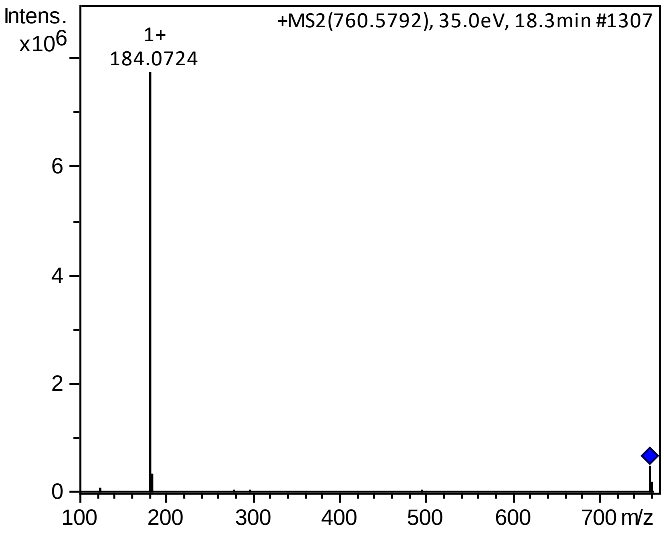

PC 16:1_22:6

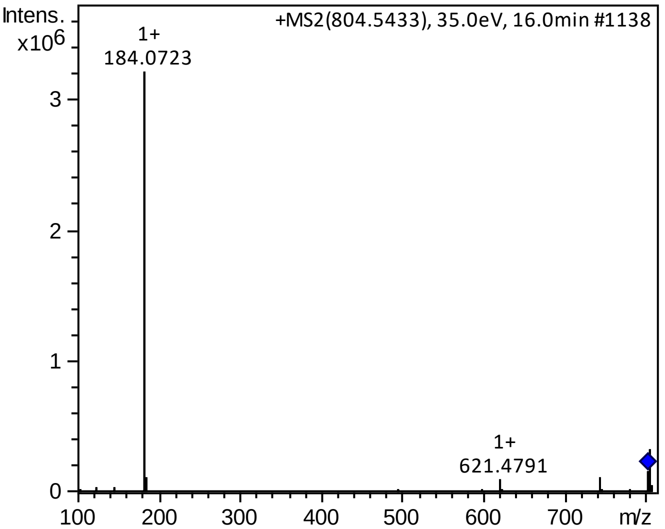

PC 18:0_18:2

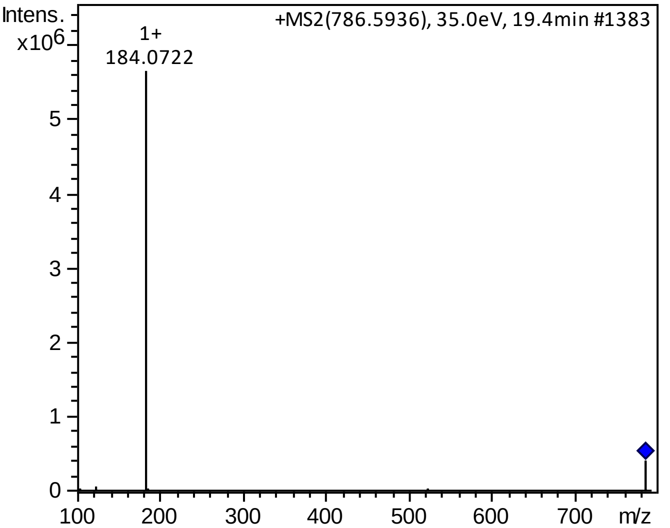

PC 18:1_18:3

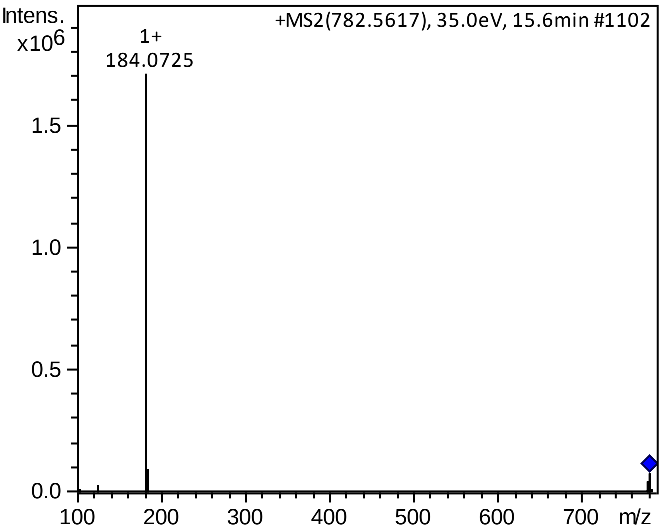

PC 18:1_20:1

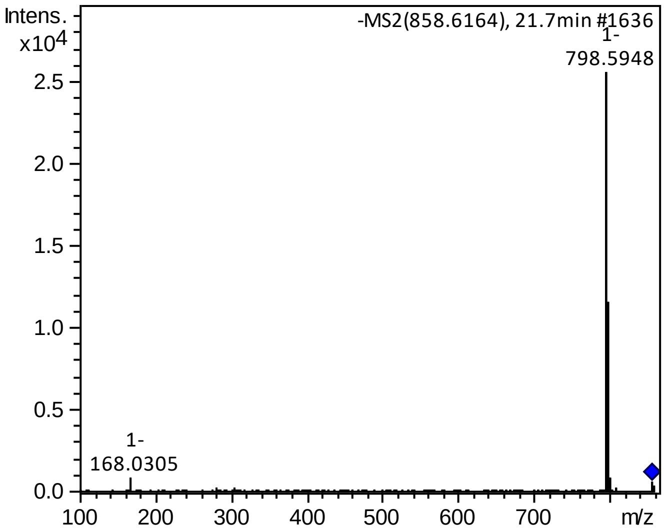

PC 18:2_18:2

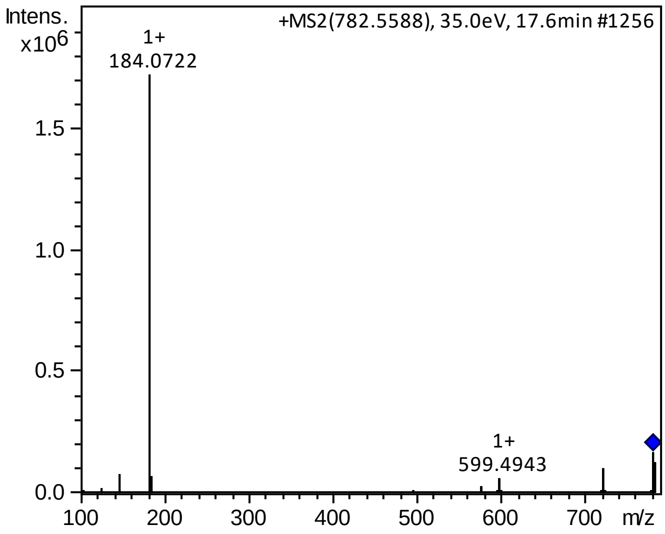

PE 18:0_20:1

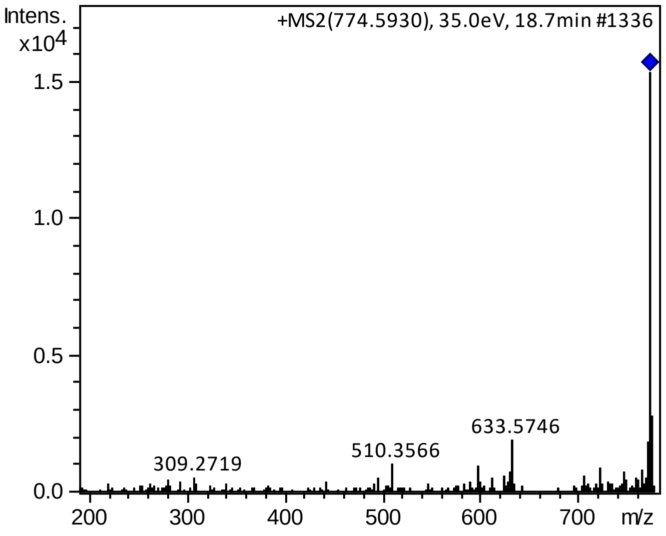

PC O-16:0/20:4

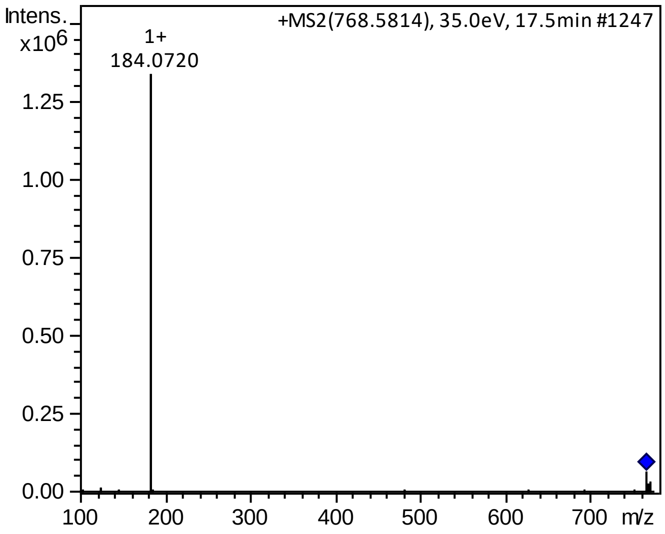

PC O-18:0/18:1

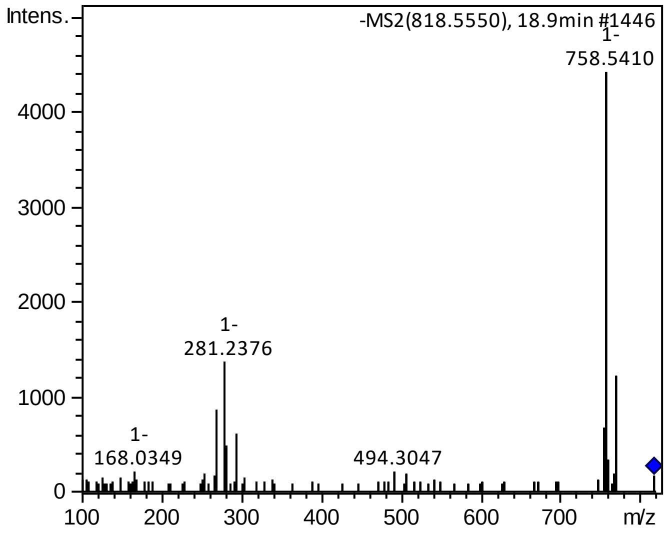

PC P-16:0/18:2

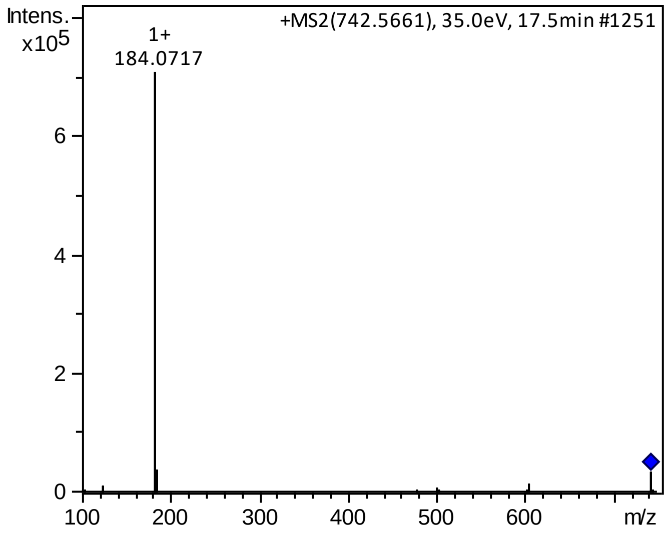

PC P-20:1/18:1

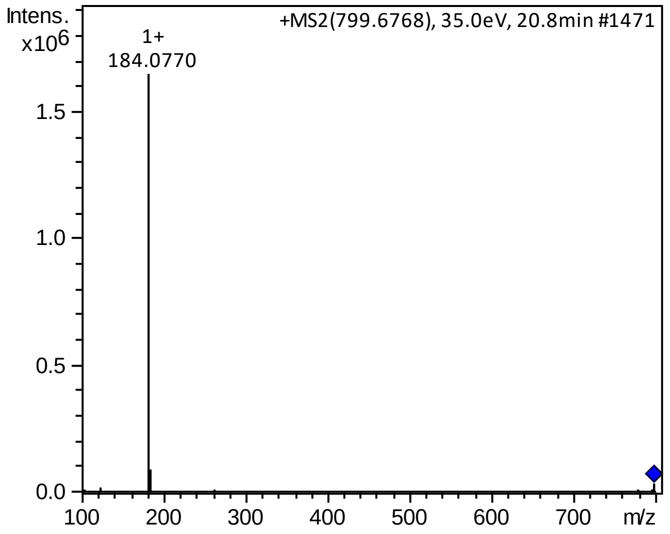

SM d18:0/22:0

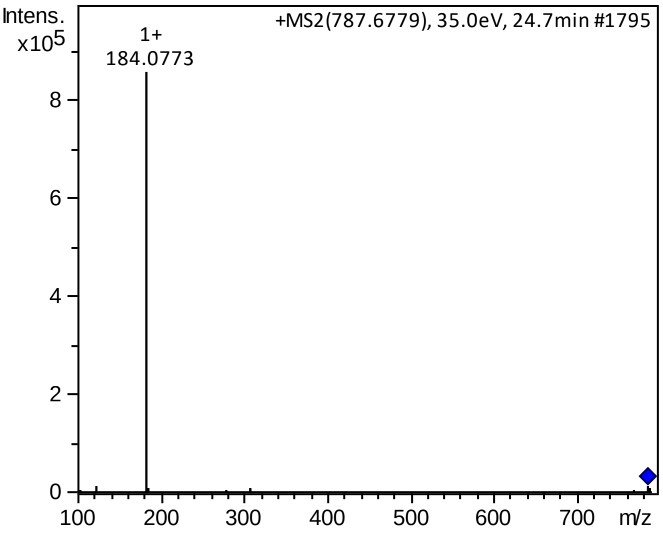

SM d18:1/18:3

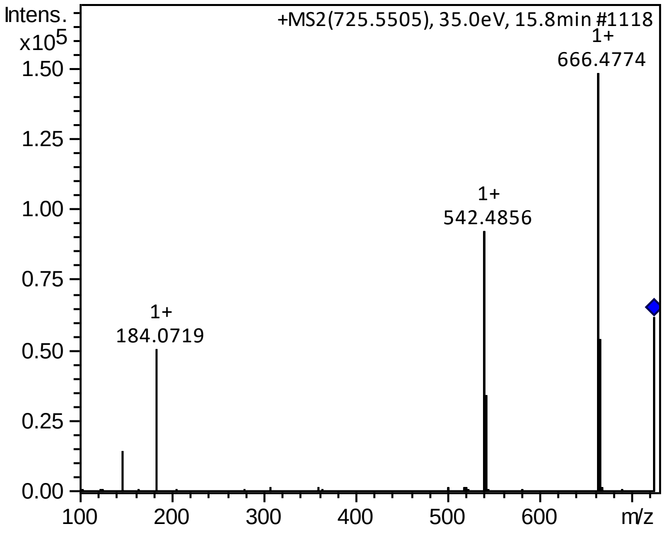

SM d18:1/24:1

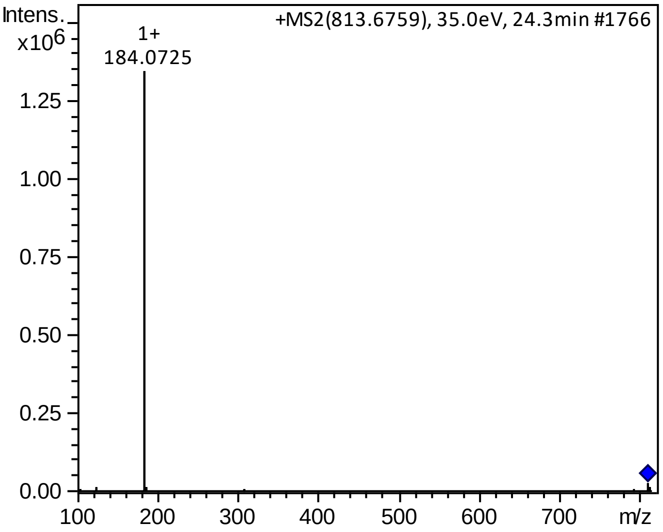

TG 14:0_16:1_18:2

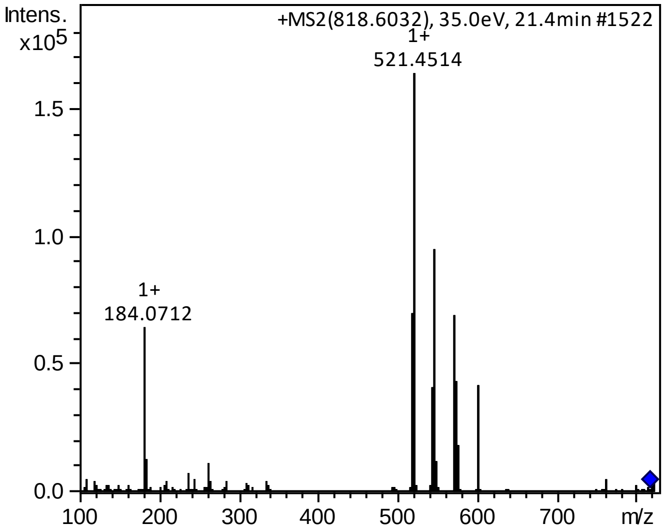

TG 16:0_16:0_18:1

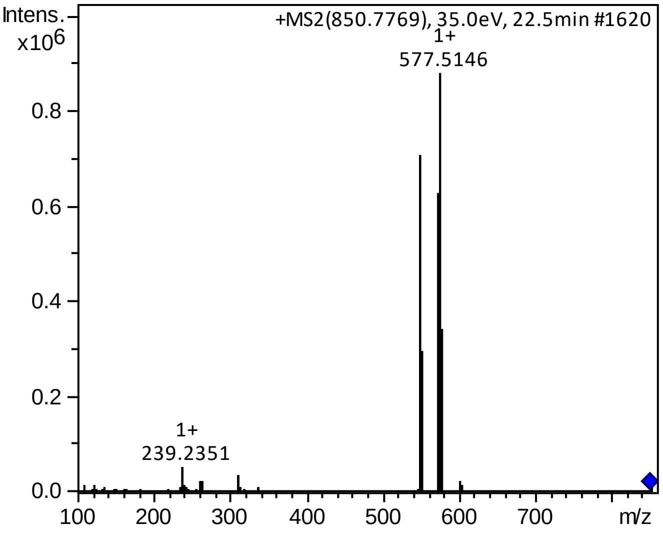

TG 16:0_16:1_18:0

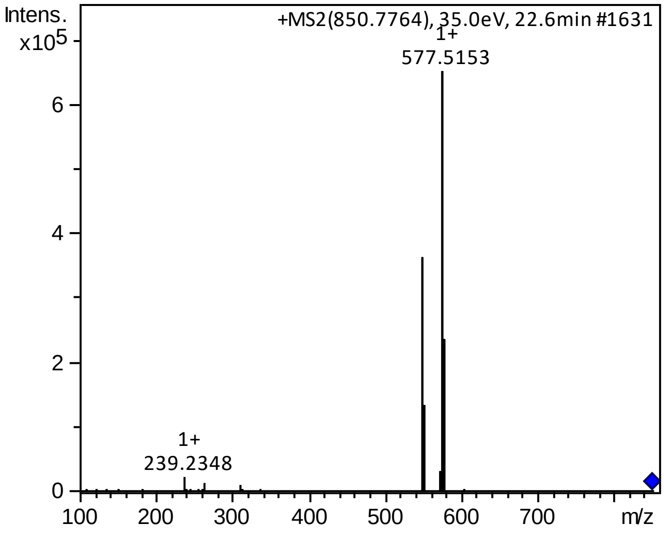

TG 18:1_18:1_20:1

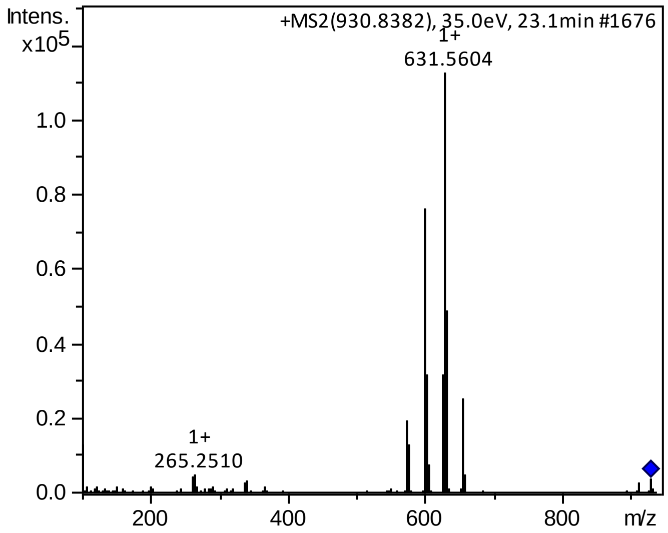

TG 18:1_22:5_8:0

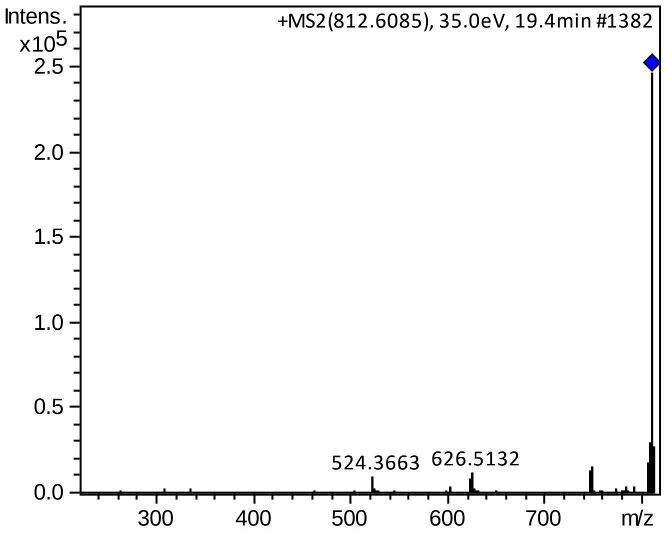

Supplement: Supplementary file 1 [file ijms-24-13787-s001.zip › Supplementary S5.pdf]
