# Supplementary figures and images for: COVID-19 Infection during Pregnancy: Disruptions in Lipid Metabolism and Implications for Newborn Health
Source: Int J Mol Sci. 2023 Sep 7;24(18):13787. doi: 10.3390/ijms241813787 (PMC10531385; doi:10.3390/ijms241813787)

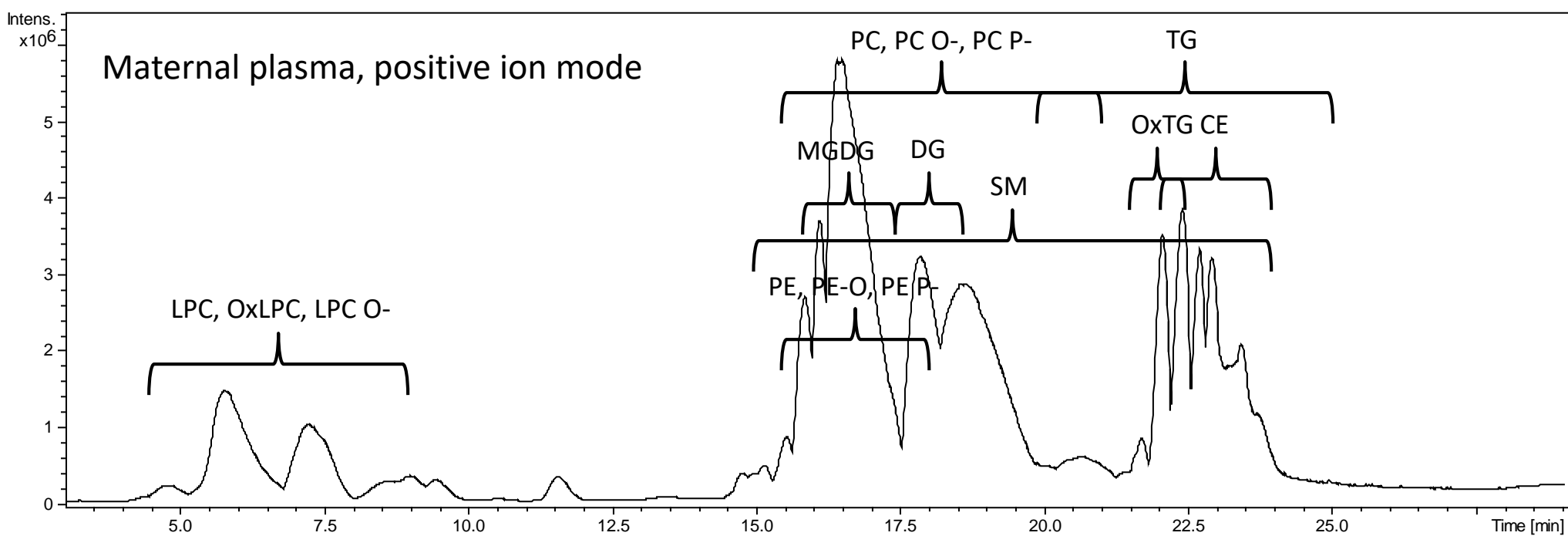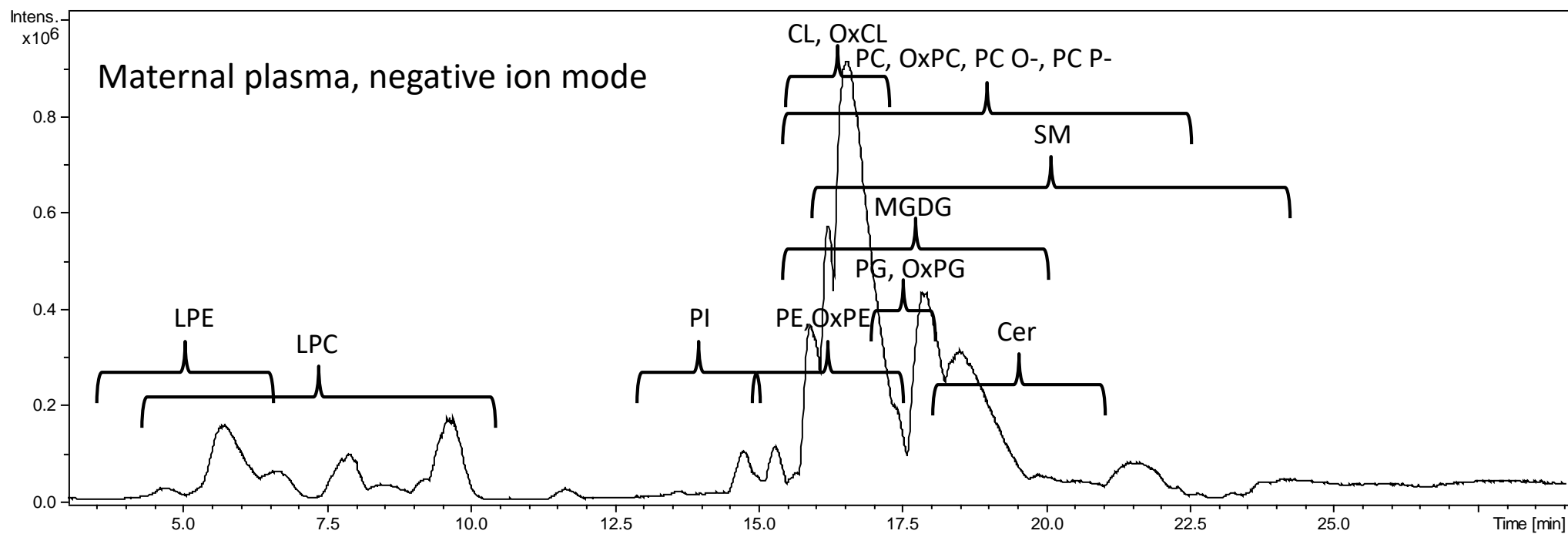

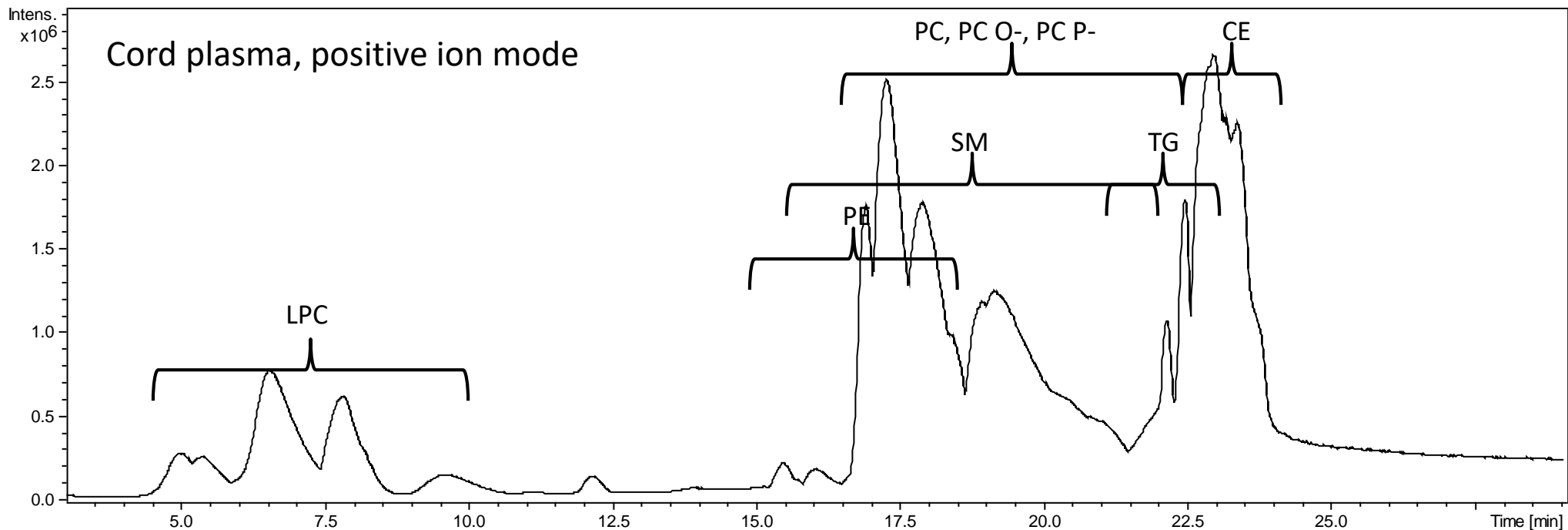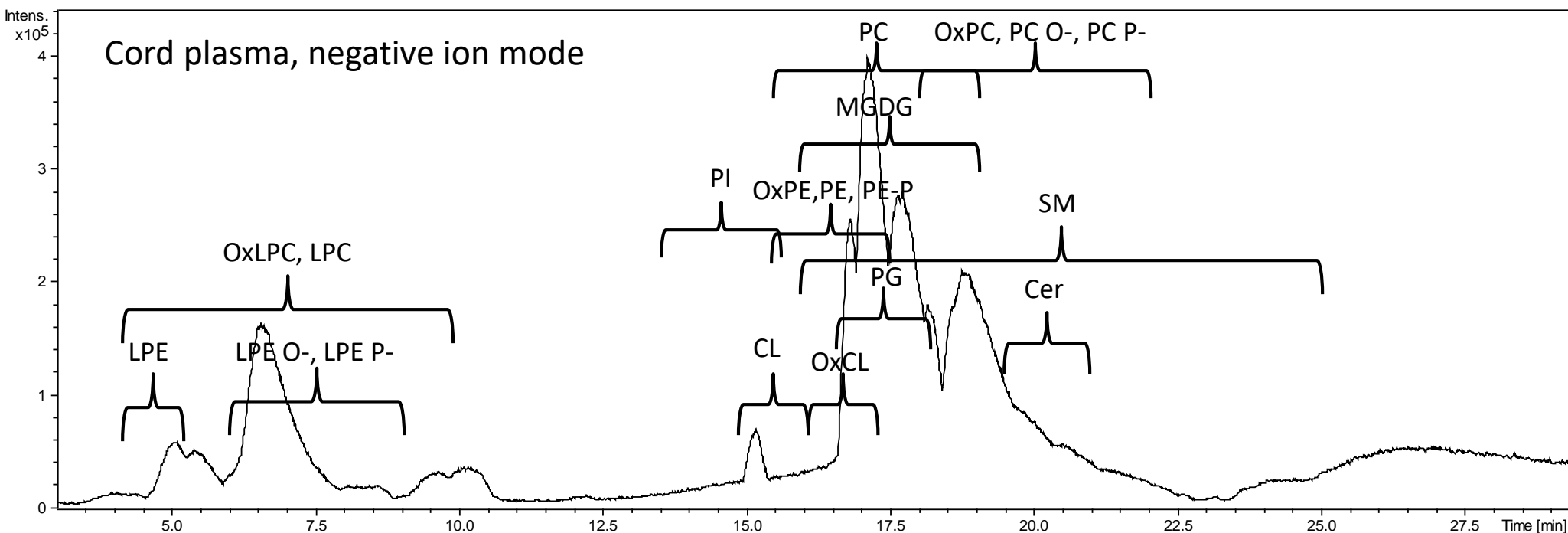

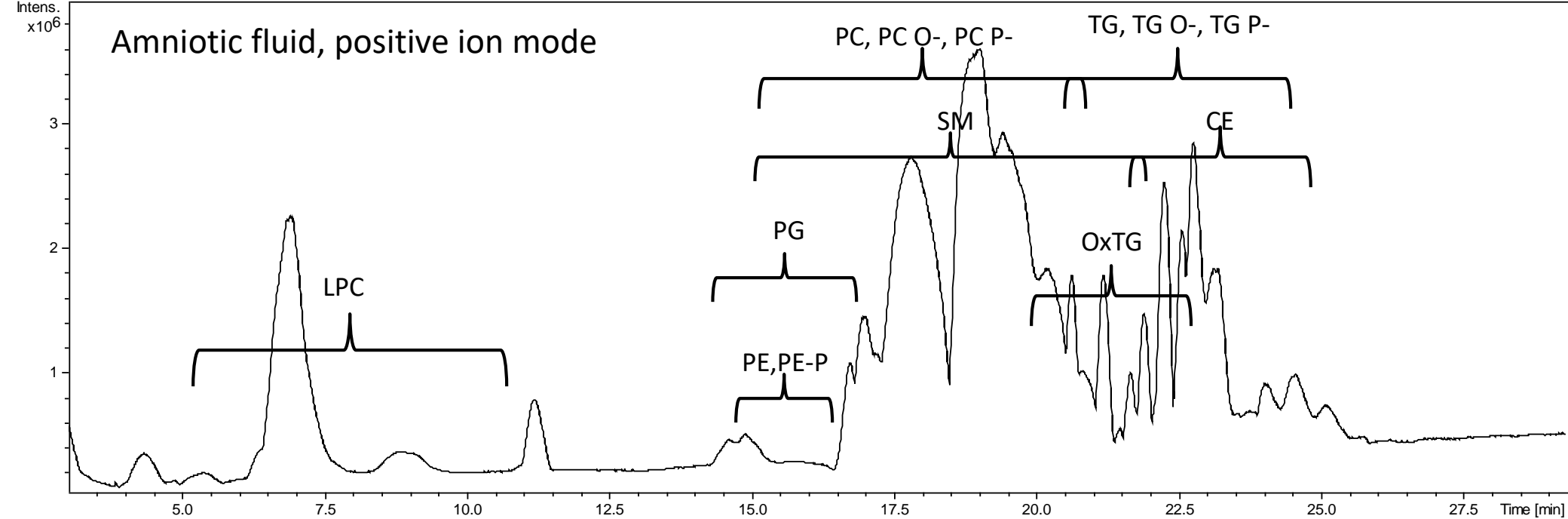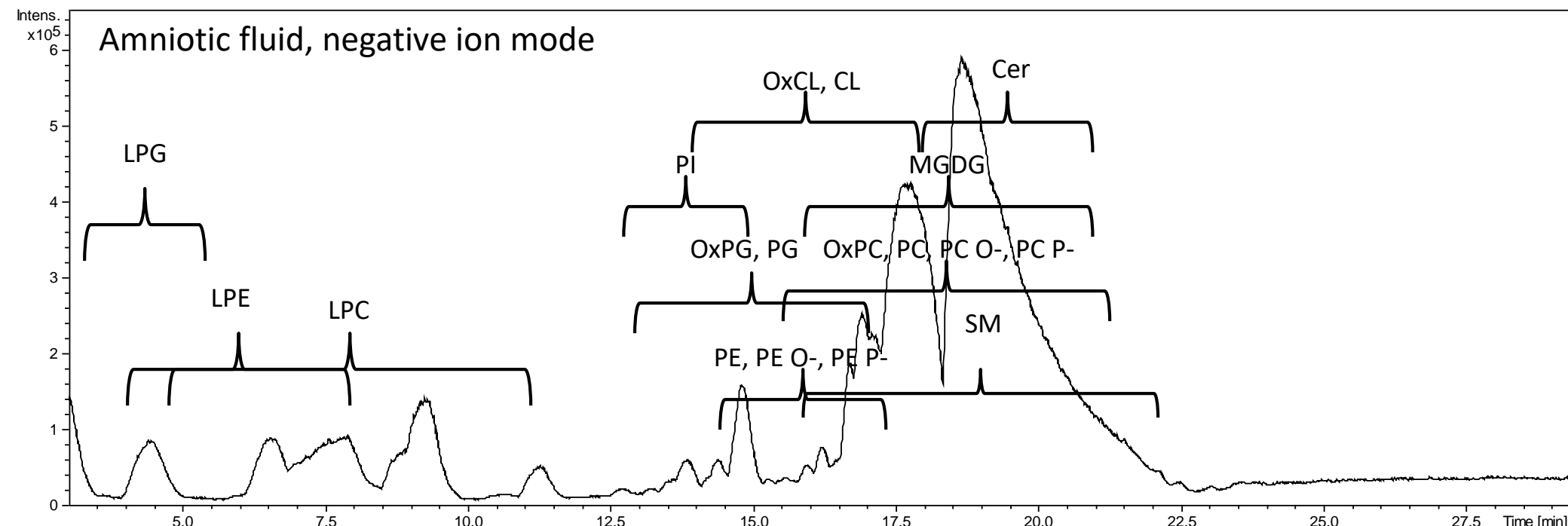

Supplement: Supplementary file 1 [file ijms-24-13787-s001.zip › Supplementary S3.pdf]
